# Conventional and genetic associations of adiposity with 1463 proteins in relatively lean Chinese adults

**DOI:** 10.1007/s10654-023-01038-9

**Published:** 2023-09-07

**Authors:** Pang Yao, Andri Iona, Christiana Kartsonaki, Saredo Said, Neil Wright, Kuang Lin, Alfred Pozarickij, Iona Millwood, Hannah Fry, Mohsen Mazidi, Yiping Chen, Huaidong Du, Derrick Bennett, Daniel Avery, Dan Schmidt, Pei Pei, Jun Lv, Canqing Yu, Michael Hill, Junshi Chen, Richard Peto, Robin Walters, Rory Collins, Liming Li, Robert Clarke, Zhengming Chen, China Kadoorie Biobank Collaborative Group

**Affiliations:** 1 Clinical Trial Service Unit, Nuffield Department of Population Health, University of Oxford, Old Road Campus, Roosevelt Drive, Oxford, OX3 7LF UK; 2 Medical Research Council Health Research Unit, Nuffield Department of Population Health, University of Oxford, Oxford, UK; 3 Peking University Center for Public Health and Epidemic Preparedness and Response, Beijing, China; 4 Department of Epidemiology and Biostatistics, School of Public Health, Peking University, Beijing, China; 5 China National Center for Food Safety Risk Assessment, Beijing, China

**Keywords:** Obesity, Body mass index, Proteomics, Mendelian randomization analysis, Drug discovery, East Asians

## Abstract

Adiposity is associated with multiple diseases and traits, but little is known about the causal relevance and mechanisms underlying these associations. Large-scale proteomic profiling, especially when integrated with genetic data, can clarify mechanisms linking adiposity with disease outcomes. We examined the associations of adiposity with plasma levels of 1463 proteins in 3977 Chinese adults, using measured and genetically-instrumented BMI. We further used two-sample bi-directional MR analyses to assess if certain proteins influenced adiposity, along with other (e.g. enrichment) analyses to clarify possible mechanisms underlying the observed associations. Overall, the mean (SD) baseline BMI was 23.9 (3.3) kg/m^2^, with only 6% being obese (i.e. BMI ≥ 30 kg/m^2^). Measured and genetically-instrumented BMI was significantly associated at FDR < 0.05 with levels of 1096 (positive/inverse: 826/270) and 307 (positive/inverse: 270/37) proteins, respectively, with FABP4, LEP, IL1RN, LSP1, GOLM2, TNFRSF6B, and ADAMTS15 showing the strongest positive and PON3, NCAN, LEPR, IGFBP2 and MOG showing the strongest inverse genetic associations. These associations were largely linear, in adiposity-to-protein direction, and replicated (> 90%) in Europeans of UKB (mean BMI 27.4 kg/m^2^). Enrichment analyses of the top > 50 BMI-associated proteins demonstrated their involvement in atherosclerosis, lipid metabolism, tumour progression and inflammation. Two-sample bi-directional MR analyses using *cis*-pQTLs identified in CKB GWAS found eight proteins (ITIH3, LRP11, SCAMP3, NUDT5, OGN, EFEMP1, TXNDC15, PRDX6) significantly affect levels of BMI, with NUDT5 also showing bi-directional association. The findings among relatively lean Chinese adults identified novel pathways by which adiposity may increase disease risks and novel potential targets for treatment of obesity and obesity-related diseases.

**Supplementary Information:**

The online version contains supplementary material available at 10.1007/s10654-023-01038-9.

## Introduction

Worldwide obesity affects about 700 million adults and the prevalence continues to increase steadily in most countries, including China [1]. The effects of obesity, or more broadly of adiposity, on metabolic traits (e.g. lipids, blood glucose, and blood pressure), cardiovascular diseases, type 2 diabetes, and certain cancers are well established [2–7]. However, substantial uncertainty persists about the aetiological role of adiposity for many other diseases and about the mechanisms linking adiposity with individual diseases.

In addition to acting as a location of energy storage, adipose tissue, whether located in the subcutaneous fat, peri-muscular fat, intra-peritoneal fat, between the internal organs, or even within the internal organs (e.g. liver), also functions as an endocrine organ [8]. Hence, adipose tissue produces hormones (e.g. leptin, oestrogen, and resistin), inflammatory biomarkers, fatty acids and adipo-cytokines [8], which can target multiple body systems and trigger the onset of multiple diseases, beyond established metabolic pathways. Moreover, it is likely that multiple novel biomarkers (e.g. circulating proteins and small molecules) associated with adiposity have yet to be identified.

The majority of druggable targets are proteins, including enzymes, protein kinases and transport proteins [9]. Systematic characterisation of large number of circulating proteins in humans has only recently become possible with the advent of high-throughput proteomic assays [9–11]. Analyses of the observational and genetic associations of plasma levels of proteins with adiposity traits have implicated novel proteins in disease aetiology and molecular pathways linking adiposity (and other risk factors) with multiple diseases [12–17]. However, the available evidence on proteomics has been constrained by studies involving relatively small numbers of proteins using targeted cardiometabolic or inflammation panels [12–14], or restricted to Western populations in whom most were overweight or obese [13–17], or used self-reported measures of adiposity [15], and often lacked concomitant genetic analyses to assess the causal relevance and direction (i.e. whether in adiposity-to-protein direction, or vice versa) of these associations [14]. Comprehensive evaluation of the associations of adiposity with protein biomarkers in Chinese adults should be particularly informative as mean adiposity levels (e.g. mean BMI of 22–24 kg/m^2^), body shape and genetic architecture differ importantly from those in Western populations.

We undertook observational and genetic analyses of adiposity with 1463 proteins in 3977 Chinese adults selected from the China Kadoorie Biobank (CKB). The main aims of the present study were to identify plasma proteins that were significantly associated with BMI and to clarify the shape, strength, and causal relevance of the observed associations. Moreover, we also used gene ontology (GO) enrichment analyses to explore whether particular classes of proteins are affected by BMI, and bi-directional MR analyses to examine whether certain proteins may also causally affect levels of BMI.

## Methods

### Study population

Details of the CKB design and methods have been previously reported [18, 19]. Briefly, 512,869 participants aged 30–79 years were recruited during 2004–2008 from 10 (5 urban, 5 rural) geographically diverse areas. At the baseline survey, participants completed an interviewer-administered laptop-based questionnaire on sociodemographic and lifestyle factors (e.g. smoking, alcohol drinking and physical activity), and medical history and medication (e.g. statin), underwent physical measurements (e.g. blood pressure, heart rate, height and weight, and waist and hip circumferences), and provided a 10 mL non-fasting blood sample (with time since last meal recorded) for long-term storage. Prior international, national and regional ethical approvals were obtained and all participants provided written informed consent for participation.

### Anthropometric measurements

Anthropometric measurements were recorded with participants wearing light clothing but without shoes, and usually to the nearest 0.1 cm or 0.1 kg. Weight was measured using a body composition analyser (TANITA-TBF-300GS; Tanita Corporation), with subtraction of weight of clothing by 0.5 kg in summer, 1.0 kg in spring/autumn and 2.0–2.5 kg in winter. BMI was calculated by weight in kilograms divided by the square of the height in metres (kg/m^2^).

### Proteomics assay

The proteomics assay was conducted among 3977 CKB participants, who had no prior history of cardiovascular diseases, no use of lipid-lowering drugs (e.g. statins) at time of sample collection, but had genome-wide genotyping data available. Participants were selected as part of a nested case-cohort study, involving 1951 incident IHD cases and 2026 randomly selected subcohort individuals (eFigure 1).

Stored baseline plasma samples from participants were retrieved, thawed, and sub-aliquoted to multiple aliquots, with one (100 µL) shipped on dry ice to the OLINK Biosciences Laboratory at Uppsala, Sweden, for proteomic analysis using a multiplex proximity extension assay. To minimize inter- and intra-run variation, the samples were randomized across plates and normalized using both an internal control (extension control) and an inter-plate control and then transformed using a pre-determined correction factor.

Details of the OLINK assay performance and validation have been reported elsewhere [10]. The LOD was determined using negative control samples (buffer without antigen). A sample was flagged as having QC warning if the incubation control deviated more than a pre-determined value (± 0.3) from the median value of all samples on the plate (but values below LOD were included in the analyses). The pre-processed data were provided in the arbitrary Normalized Protein eXpression (NPX) unit on a log2 scale.

The present analyses included a total of 1472 proteins, including 3 (IL6, IL8 and TNF) that were replicated in all four individual panels, resulting in 1463 unique proteins (eTable 1). The distributions of some proteins were skewed (eFigure 2), with relatively low number of QC warnings per protein among all samples (e.g. 106 proteins had QC warnings involving 4.0% of all samples: eTable 2), which were included in the main analyses.

### Genotyping and genetic instruments for BMI

Genotyping was conducted using a custom-designed 800 K-SNP array (Axiom [Affymetrix]) for ~ 100,000 CKB participants which passed quality control (overall call rate > 99.97% for all variants), including a population-based sample of ~ 76,000 participants who were randomly selected from the overall cohort, from whom the sub-cohort of 2026 individuals was selected [20]. BMI GS was derived using loci associated at genome-wide significance in sex-combined trans-ancestry GWAS in CKB and UKB: dosages of 816 variants with minor allele frequency ≥ 0.01 associated with BMI, respectively, were weighted according to their effect sizes in UKB [21]. The BMI-GS (F-statistic: 152) was strong instrument (with 3.7% of variance explained) and not associated with confounders such as smoking or alcohol consumption (eTable 3). Genetic instruments for bi-directional MR were *cis*-pQTLs from GWAS for each protein.

### Statistical analysis

The prevalence or mean values of baseline characteristics were calculated by BMI quintiles, standardised to the age (5-year groups), sex and study area structure of the cases and subcohort. Plasma protein levels were standardized (i.e. values of each protein were divided by their SD) and analysed as continuous variables. In observational analysis, linear regression was used to assess the associations of BMI with protein biomarkers, adjusted for age, age^2^, sex, study area, fasting time, ambient temperature, plate ID and case-subcohort ascertainment. For each biomarker, adjusted differences and 95% CIs associated with 1-SD higher levels of adiposity were estimated.

In genetic (i.e., MR) analysis, we related the genetically-instrumented BMI with proteins using the 2-stage least squares estimator method. First, the associations between the BMI GS and BMI measures were examined using linear regression, adjusting for age, age^2^, sex, study area, fasting time, ambient temperature, case-subcohort ascertainment and the first 12 national principal components. Second, the associations of the resulting predicted BMI values with proteomics measurements were examined using linear regression with the same adjustments (including plate ID) except principal components. We calculated the genetically-instrumented associations per 3.6 kg/m^2^ higher BMI (corresponding to 1-SD higher levels in the observational analyses) of measured proteins levels, to permit comparisons with the observational analyses. To replicate the main study findings, we also undertook separate observational and genetic analyses in UKB, involving the same 1463 OLINK Explore proteins in about 50,000 participants. [16]

To examine the shape of the associations in observation analyses, adjusted means of individual proteins were calculated within each of BMI quintiles using multiple linear regression and then plotted against mean BMI within each of quintiles. Similarly, in genetic analyses, we undertook non-linear MR analyses by stratifying GS-free BMI based on its quintiles. GS-free BMI was calculated as the residuals from the regression of BMI on GS, centered on the overall population mean BMI (23.9 kg/m^2^) [22]. We then calculated causal estimates for each stratum using the ratio method. The overall estimate of the BMI-GS association was used as the denominator, with the numerator being the estimate from the association of GS with each protein, within each of the GS-free BMI quintiles. The piecewise linear method was used to estimate the mean of each GS-free BMI stratum, using the causal estimate as the slope of the line in each stratum. Each line segment begins where the previous segment ends. The intercept was set to the population mean BMI. The CIs were estimated by bootstrapping the associations of the GS with protein biomarkers in samples of each GS-free BMI strata, and the χ^2^ values for trend and quadratic test were calculated for the causal estimates across the GS-free BMI startum for each protein.

In sensitivity analyses, we (i) restricted analyses to subcohort participants only; (ii) excluded values with QC warnings; (iii) adjusted for additional covariates (e.g. education, smoking, alcohol drinking, and physical activity); (iv) excluded individuals with prior diseases. We also performed sex-specific analyses to check the consistency of results between men and women.

For proteins that passed Bonferroni-corrected threshold in the genetic analyses (adiposity-to-protein direction), we conducted GO and KEGG enrichment analyses using clusterProfiler (v.4.2.2) [23], to determine which biological functions or processes were significantly enriched based on hypergeometric tests.

In GWAS analyses of CKB participants, pQTLs were determined using the COJO method [24], with a threshold at *P* < 5 × 10^–8^ for statistical significance. Moreover, for all proteins with available *cis*-pQTLs (± 500 kb around the encoded gene region), a two-sample bi-directional MR was conducted using (i) *cis*-pQTLs obtained from GWAS of CKB, with lookups separately in BBJ (n = 173,430) [25] and (ii) *cis*-pQTLs obtained from GWAS of UKB, with lookups in GIANT with (n ∼ 700,000) [26] or without (n ~ 210,000) UKB participants [27]. Both analyses used the two-stage least squares estimation and Wald ratio methods. [28, 29] For those proteins showing significant associations in 2SMR, the causal direction of each extracted SNP to the levels of protein and BMI was tested using MR Steiger filtering [30]. For proteins of interest, we also undertook colocalisation analyses using coloc (v5.2.1) to investigate whether they shared the same causal variants with BMI, and explored the protein–protein interaction using the STRING database (v11.5). Protein expression database of GTEx (v8) [31] was screened to examine the tissue-specific role of the causal proteins in obesity, and tissues involved in energe metabolism or endocrine control of food intake were selected. We further searched PhenoScanner (v2) and GWAS Catalog (v1.0.2) for associations of *cis*-pQTLs from both CKB and UKB with a range of phenotypes using a *P* value threshold of 5 × 10^–8^.

All statistical analyses were performed using R version 4.1.2. Significance thresholds used Benjamini–Hochberg FDR or the more stringent Bonferroni-corrected thresholds (0.05/1463) to correct for multiple testing.

## Results

Of the 3977 participants studied, the mean (SD) baseline age was 57.3 (11.6) years, and the mean BMI was 23.9 (3.3) kg/m^2^, with 6% being obese (i.e. BMI ≥ 30 kg/m^2^). Participants with higher BMI had higher levels of blood pressure, were more likely to be urban residents and women, and less likely to be smokers (in men only) (Table 1). These associations were broadly similar in IHD cases and subcohort participants, although IHD cases had higher mean levels of blood pressure than subcohort participants (eTable 4).

**Table 1:** Baseline characteristics of participants by quintiles of measured baseline BMI in CKB

Characteristics^a^	Quintiles of BMI, kg/m^2^					All (n = 3977)
	Q1 (n = 796)	Q2 (n = 789)	Q3 (n = 812)	Q4 (n = 785)	Q5 (n = 795)	
*Age and socioeconomic factors*						
Age, years (SD)	58.8 (15.1)	56.2 (12.5)	56.3 (11.9)	56.9 (12.1)	56.9 (12.8)	57.3 (11.6)
Women, %	52.9	51.2	53.0	51.0	62.6	53.7
Urban, %	28.1	39.2	53.0	59.6	65.6	48.8
≥ 6 years of education, %	43.4	45.8	46.7	46.4	44.4	45.1
*Anthropometry and blood pressure, mean (SD)*						
BMI, kg/m^2^	19.2 (1.3)	21.9 (0.6)	23.7 (0.5)	25.7 (0.6)	29.2 (1.9)	23.9 (3.3)
Waist circumference, cm	70.5 (5.1)	77.2 (5.5)	81.6 (5.4)	86.6 (5.1)	94.1 (9.5)	81.9 (9.1)
SBP, mmHg	129.8 (21.8)	133.6 (19.3)	138.8 (22.0)	142.1 (21.9)	146.5 (23.7)	138.3 (22.0)
Fasting time	4.4 (4.9)	4.7 (4.3)	4.9 (4.1)	4.7 (3.8)	4.3 (3.5)	4.7 (4.1)
Lifestyle factors						
*Ever regular smoker*, %						
Men	82.8	75.4	73.2	70.0	75.8	75.0
Women	5.6	7.4	6.2	4.0	5.1	5.8
*Regular alcohol consumption*, %						
Men	34.6	35.4	36.4	36.0	31.0	34.6
Women	3.2	2.5	2.0	4.1	3.6	3.0
Physical activity, MET-h/day (SD)	17.6 (11.2)	17.4 (10.5)	17.2 (12.1)	17.4 (10.3)	16.0 (10.0)	17.3 (10.7)
*Medical history and health status*^b^, %						
Self-rated poor health	13.2	16.5	16.6	18.7	18.0	16.6
Diabetes	5.9	7.9	11.1	13.3	17.7	11.2
Chronic kidney disease	1.0	1.0	1.6	1.7	1.5	1.4
Cancer	0.8	0.8	0.4	0.4	0.9	0.6

*SD* standard deviation; *BMI* body mass index; *SBP* systolic blood pressure; *MET* metabolic equivalent of task

^a^Adjusted for age, sex and study area, as appropriate

^b^Based on self-report, while for diabetes, those with screen-detected cases at baseline were also included

### Observational associations of BMI with proteins

**Fig. 1:**
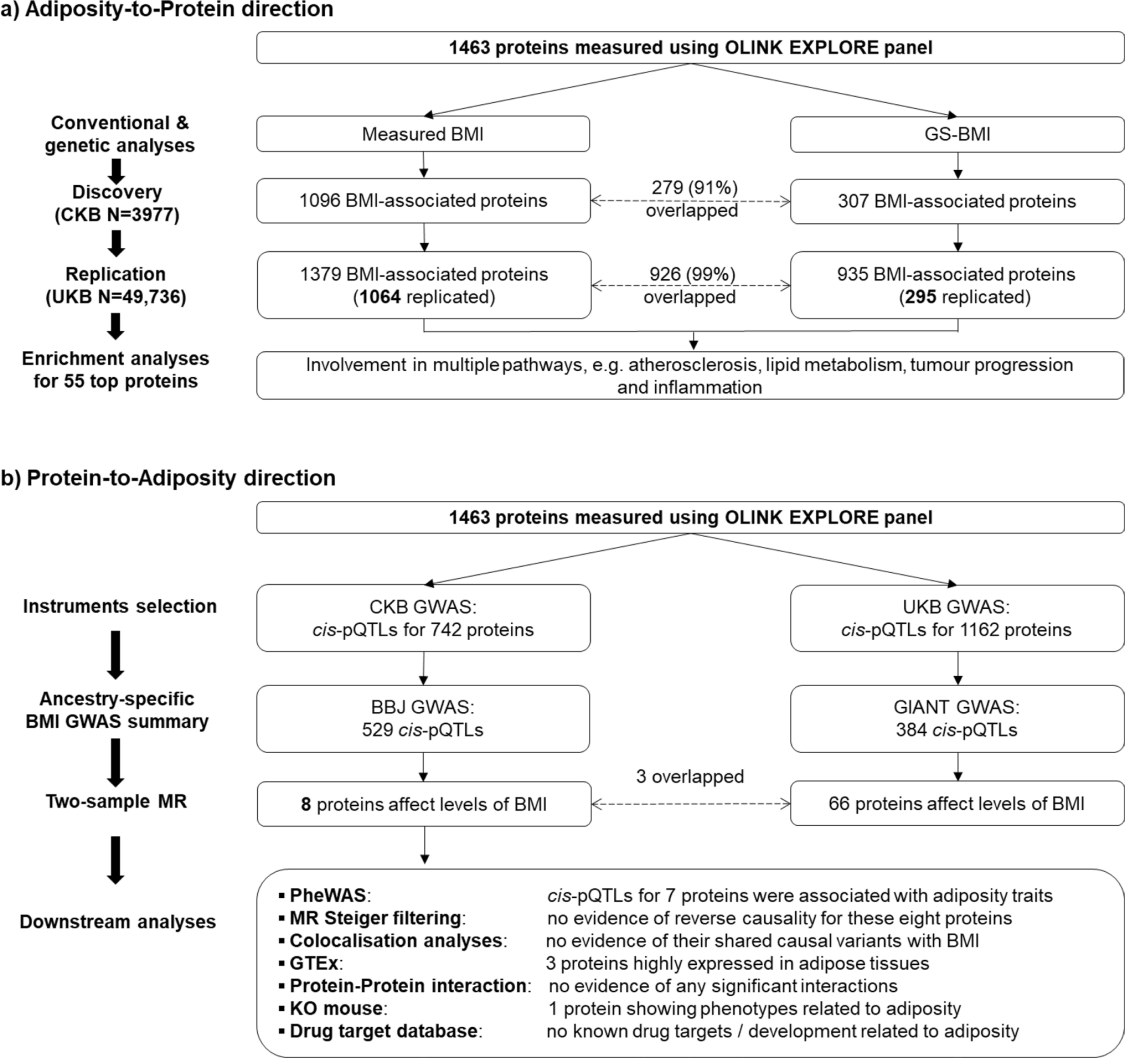
Overview of analytic approaches and key findings

**Fig. 2:**
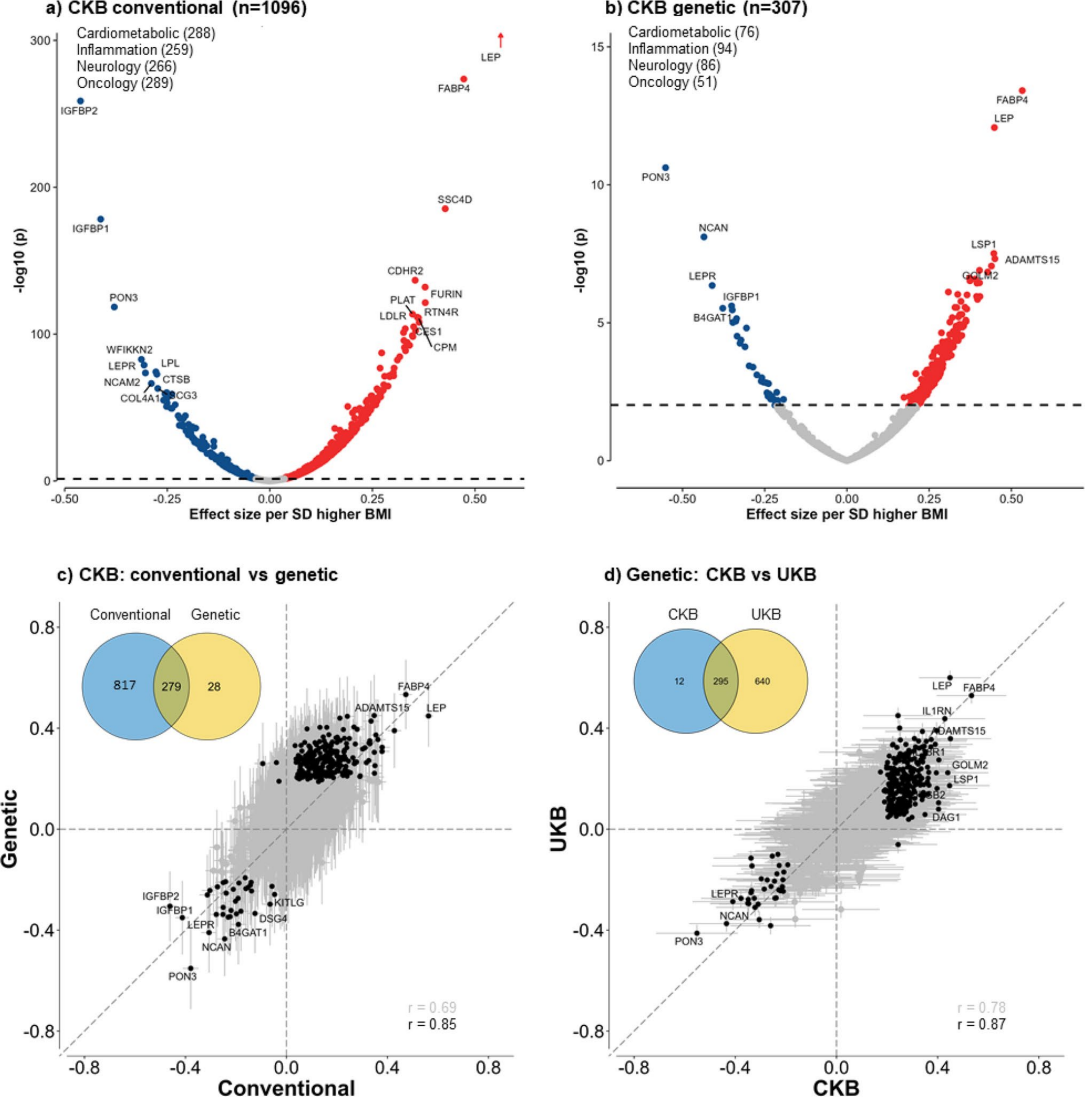
Associations of 1-SD higher BMI with 1463 proteins in conventional and genetic analyses in CKB and comparisons of genetic associations between CKB and UKB. Analyses were adjusted for age, age square, sex, study area, fasting time, ambient temperature, ascertainment status, plate ID, and the first 12 PCs (for genetic analyses only). The dotted lines in **a** and **b** indicate multi-testing adjusted threshold for statistical significance with red dots showing significant positive associations and blue dots showing significant inverse associations, with names given for certain selected proteins. The solid black dots in **c** and **d** are proteins significantly associated with BMI in both conventional and genetic analyses in CKB (left panel) or in both CKB and UKB (right panel), with names given for certain selected proteins

Overall, BMI was significantly associated at FDR < 0.05 with plasma levels of 1096 proteins, with 826 being positive and 270 inverse (Fig. 1a and eFigure 3). After applying Bonferroni significance threshold, 798 (625 positive, 173 inverse) proteins remained significantly associated with BMI. Almost all associations of BMI with individual proteins were linear throughout the full ranges of BMI examined (eFigure 4), although the strength of the associations varied, with effect sizes per SD higher BMI ranging from 0.01 to 0.55 for positive associations and from − 0.45 to − 0.01 for inverse associations (Fig. 2a). The proteins showing the strongest positive associations with BMI were leptin, FABP4, SSC4D and CDHR2, and FURIN, while the proteins showing strongest inverse associations were IGFBP2, IGFBP1, PON3, WFIKKN2 and LEPR. The results for BMI with all individual proteins are shown in eTable 5.

In sensitivity analyses restricted to subcohort participants, BMI was associated at FDR < 0.05 with 984 proteins (eFigure 5), with > 97% overlapping with the main analyses and all were directionally consistent. Moreover, the overall or leading panel-specific proteins were identical to those in the main analyses. Similarly, the results were not materially altered by additional exclusion of individuals with (i) prior history of diabetes, kidney disease or cancer, or (ii) QC warnings in particular assays, or (iii) by additional adjustment for other covariates (eTable 6).

In sex-specific analyses of all participants, 921 and 970 proteins were significantly associated at FDR < 0.05 with BMI in men and women (Pearson’s correlation r = 0.87), respectively. There were 786 overlapping proteins between men and women and with exception of nine proteins, all the associations were directionally consistent (eFigure 6).

### Genetic associations of BMI with proteins

**Fig. 3:**
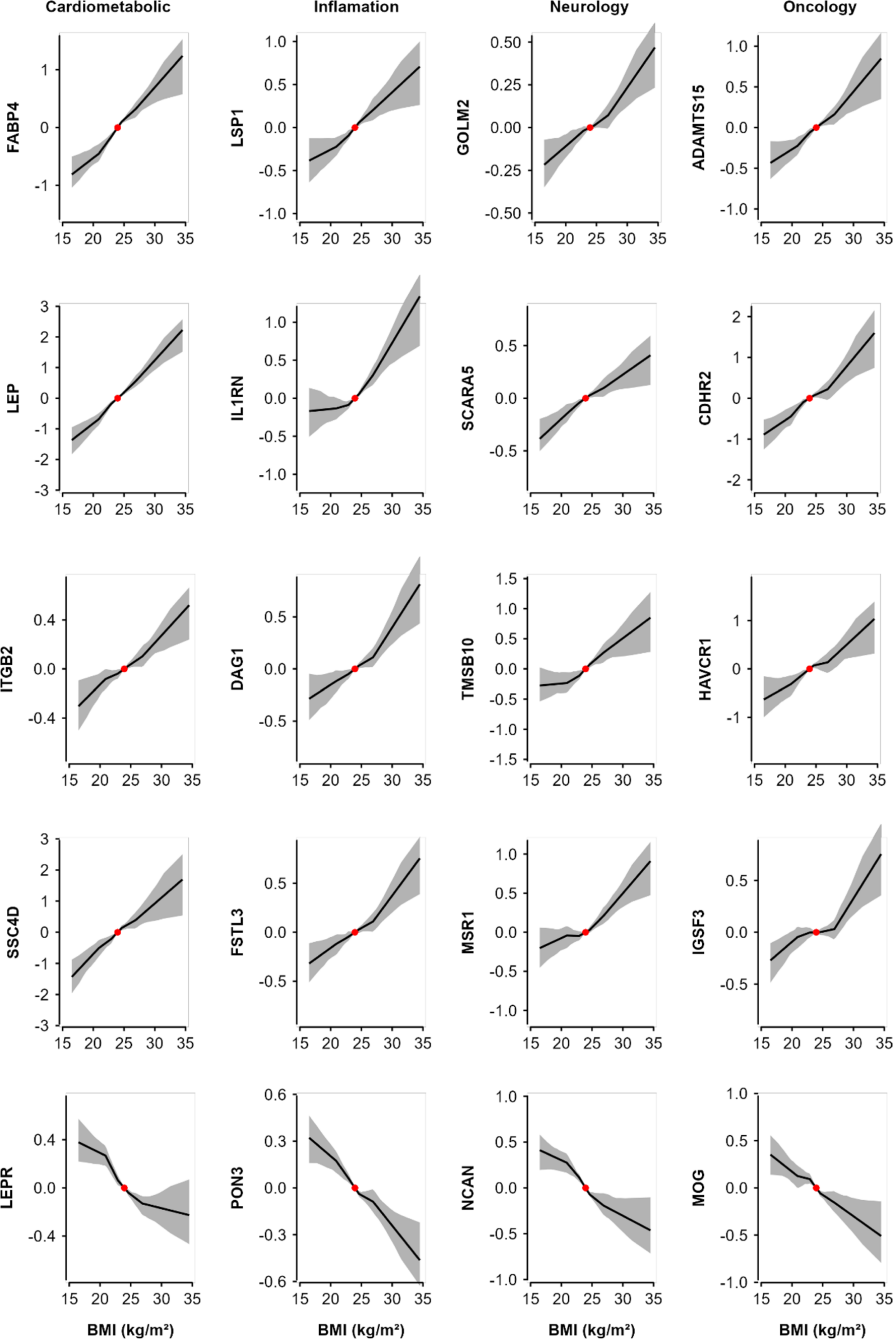
Genetic associations of BMI with 20 selected proteins, by OLINK panel. Non-linear MR analyses was used to investigate the shape of the genetic associations. Within each panel, top 5 (4 positive and 1 inverse) BMI-associated proteins were included. Piecewise linear method was used to calculate the estimates (adjusted for age, age squared, sex, and study area [ten groups], ascertainment, plate ID, and 12 national PCs). Each line segment begins where the previous segment finished (black lines) and the intercept was set to the population mean BMI (red dot). The 95% CI are represented by the shaded patterns. The length of y-axis represents approximately ± 2 SD from the mean of the corresponding protein

In genetic analyses, 307 (270 positive, 37 inverse) proteins were significantly associated at FDR < 0.05 with genetically-derived BMI (Fig. 1), compared with 55 (43 positive, 12 inverse) proteins when applying Bonferroni correction. Of these 307 proteins, 279 (91%) also showed significant associations in the observational analyses (Fig. 2b), with directionally consistent results for all (242 positive, 34 inverse) except three proteins (CKMT1A_CKMT1B, MMP12 and SLAMF7; all inverse in observational but positive in genetic analyses). There was a strong correlation between the beta coefficients from observational and MR estimates, with Pearson’s correlation coefficients of 0.69 (0.66–0.72) for BMI, increasing to 0.85 (0.81–0.88) after removing all non-significant proteins. Moreover, the associations were broadly linear throughout the range of BMI examined (P_trend_ < 0.05) (Fig. 3). Conversely, among the 1156 proteins without significant linear genetic associations with BMI, 91 (7.9%) showed evidence of quadratic associations with BMI (eFigure 7), although none of them were significant after multiple testing adjustment.

The magnitude of the genetic associations per 1 SD higher predicted BMI ranged from 0.01 to 0.60 for positive associations and from − 0.55 to − 0.01 for inverse associations. The proteins showing the strongest positive associations with BMI were FABP4, followed by leptin, GOLM2, LSP1 and ADAMTS15 (Fig. 2b and eFigure 8). The proteins showing strongest inverse associations with BMI were PON3, followed by NCAN, LEPR, B4GAT1 and CHGB. The MR results for all individual proteins are shown in eTable 7.

In sensitivity analyses confined to the subcohort participants, there were fewer (83) significant associations (eFigure 9). Of these 83 proteins, 80 (96%) overlapped with the main results, all were directionally consistent (57 positive, 23 inverse). Moreover, the overall top proteins profile were identical to those in the main analyses.

In sex-specific analyses, 59 and 72 proteins were significantly associated at FDR < 0.05 with genetically-derived BMI in men and women, respectively. There were 18 overlapping proteins, with no discrepancies in the direction of the associations (13 positive, 5 inverse) but somewhat greater effect sizes in men than in women (a Pearson’s correlation r of 0.56 [0.52–0.59] between their beta coefficients) (eFigure 6).

### Replication analyses in the UK Biobank

In replication analyses of 49,736 UKB participants [16] we found 1379 (94%) proteins were significantly associated at FDR < 0.05 with BMI (mean 27.4 kg/m^2^) in conventional observational analyses (Fig. 1). Of these 1379 proteins, 1064 (97%; 1064/1096) were also significantly associated with BMI in CKB, with a Pearson correlation between the effect sizes of the overlapping proteins of 0.86 (0.84–0.87). In genetic analyses, 935 proteins showed significant associations with genetically-derived BMI (Fig. 1), which replicated most of the proteins identified in CKB (96%; 295/307; Fig. 2d), with a high correlation (0.88; 0.84–0.90) between the effect sizes of the overlapping proteins.

### Enrichment analyses

**Fig. 4:**
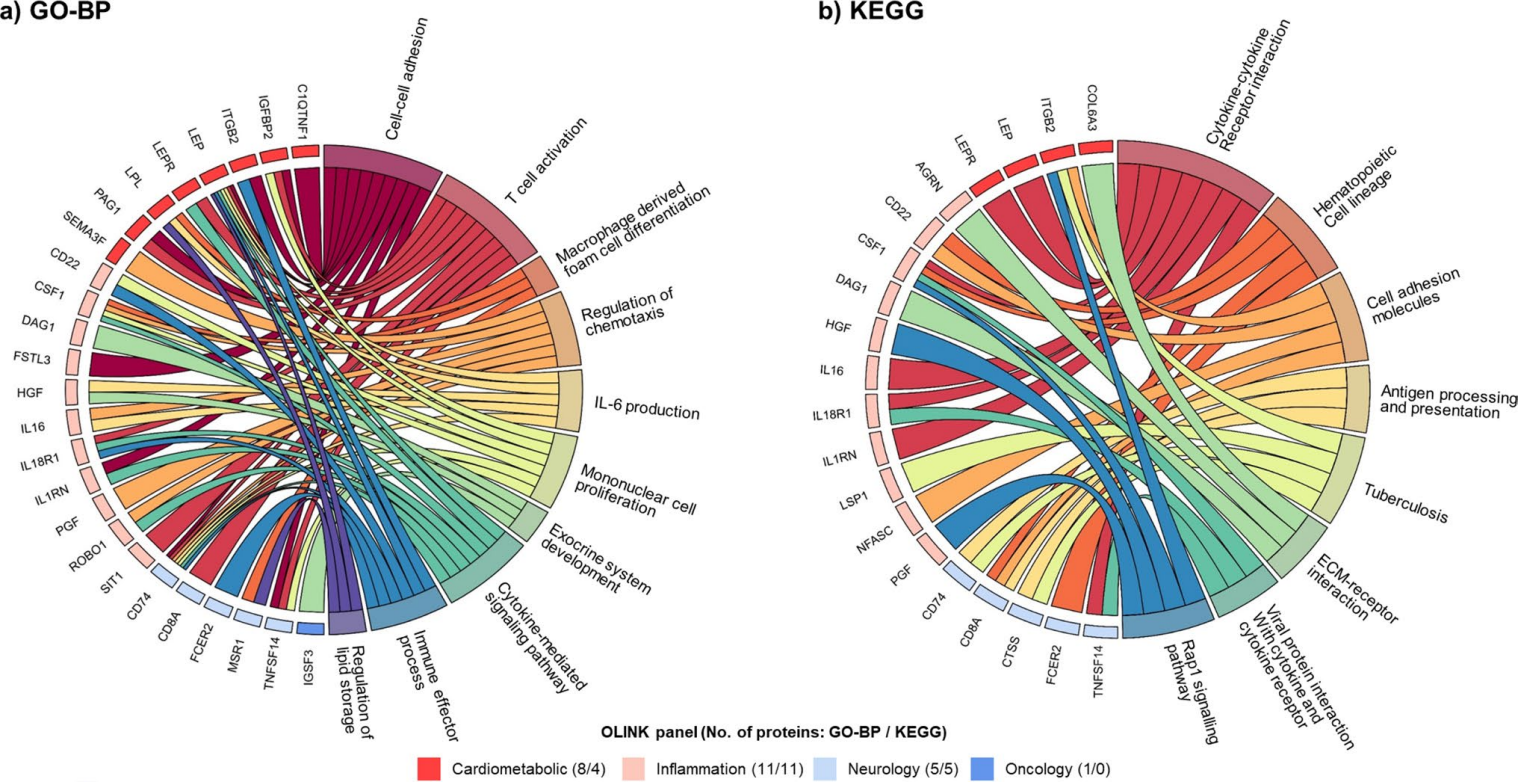
Chord diagrams of enriched **a** GO biological process terms and **b** KEGG pathways for proteins causally affected by BMI. Enrichment analyses were conducted for 55 proteins that passed Bonferroni-corrected threshold in the genetic analyses (adiposity-to-protein direction), using **a** GO-BP and **b** KEGG enrichment analyses. The right semicircle represents the names of **a** top 10 GO terms and **b** 8 significant KEGG pathways, and the left semicircle are proteins that are significantly associated with any of the GO terms or KEGG pathways. Proteins were ordered by OLINK panel, and the numbers in brackets represent the number of proteins involved in the GO terms or KEGG pathways

In enrichment analyses of top 55 BMI-associated proteins that passed Bonferroni-corrected threshold in CKB, there was strong evidence of GO enrichment for proteins in biological processes related to atherosclerosis (e.g. macrophage derived foam cell differentiation, lipid metabolism), inflammation (IL-6 production), immune function (e.g. T cell activation, mononuclear cell proliferation, immune effector process), and other biological processes (e.g. cell–cell adhesion, exocrine system development, cytokine-mediated signaling pathways; Fig. 4a). eTable 8 provide details of all significantly enriched biological process terms for BMI-related proteins beyond the top 10 terms. In sensitivity analyses comparing all 1463 OLINK proteins to all proteins with annotations, a total of 547 significant terms were identified (at FDR < 0.05), but their relative importance differed from those in the main analyses (eTable 9). In similar analyses using KEGG method (Fig. 4b and eTable 10), a total of eight pathways were annotated, including those related to tumour progression (e.g. ECM-receptor interaction, Rap1 signalling pathway), immune function (e.g. viral protein interaction with cytokine and cytokine receptor), and cell proliferation, movement and adhesion (e.g. cell adhesion molecules).

### Bi-directional MR analyses

In CKB, *cis*-pQTL were identified in GWAS for 742 of the 1463 proteins, which were used in further bi-directional two-sample MR analyses (Fig. 1b). In two-sample MR of CKB and BBJ, eight proteins (ITIH3, LRP11, SCAMP3, NUDT5, OGN, EFEMP1, TXNDC15, and PRDX6) were significantly associated with BMI (i.e. in protein-to-BMI 33direction) after correction for multiple testing, with NUDT5 also showing bi-directional association (Table 2). Moreover, independent two-sample MR analyses involving 384 *cis*-pQTLs identified in UKB GWAS for these same proteins and GIANT replicated associations for three proteins (ITIH3, OGN and TXNDC15). One protein (ITIH3) was also replicated in two-sample MR using earlier GIANT datasets without UKB. In sensitivity analyses using MR Steiger test, there was no evidence of reverse causality for these eight proteins, nor evidence of any significant interactions among them. In colocalisation analyses, there was no strong evidence (Posterior Probability H4 < 0.8) of shared causal genetic variants of these eight proteins with BMI.

**Table 2:** Genetic effect estimates, PheWAS results, tissue expression, and relevant drug targets of eight proteins showing genetic effects on BMI

Protein	Gene name	SNP Information			CKB *cis*-pQTL		Two-sample SMR^b^		CKB Obs. results^d^		UKB replication^e^	PheWAS associations^f^	Levels of expression^g^					Drug target
		rsID	EA^a^	EAF^a^	Beta (SE)	*P*	Beta (SE)^c^	*P*	Beta (SE)	*P*			Adipose	Brain	Liver	Pancreas	Intestine	
ITIH3	*ITIH3*	rs2286797	G	0.76	0.73 (0.033)	7.9E − 107	− 0.026 (0.006)	9.9E − 06	− 0.191 (0.016)	4.0E − 33	√	BMI (↓)	+	+	+++	+	+	–
LRP11	*LRP11*	rs9688902	T	0.72	0.662 (0.03)	4.7E − 113	0.03 (0.007)	8.5E − 06	0.126 (0.016)	6.4E − 16	–	Hand grip strength (↓), BMI (↑)	++	++	+	+	+	–
SCAMP3	*SCAMP3*	rs4971072	A	0.27	0.185 (0.026)	1.0E − 12	0.115 (0.027)	2.7E − 05	0.159 (0.015)	1.5E − 24	–	BMI (↑)	++	++	++	++	++	–
NUDT5^h^	*NUDT5*	rs7100710	A	0.47	0.154 (0.023)	2.4E − 11	-0.122 (0.029)	3.8E − 05	0.159 (0.015)	1.8E − 25	–	**BMI** (↓)	++	+	++	+	++	–
OGN	*OGN*	rs7023004	G	0.26	0.466 (0.039)	1.5E − 80	0.033 (0.009)	3.2E − 04	0.092 (0.018)	2.6E − 07	√	**Impedance of arm/leg/whole body** (↓), **height** (↓)	+++	+	+	+	+	–
EFEMP1	*EFEMP1*	rs58680090	A	0.75	0.166 (0.028)	4.3E − 09	0.11 (0.031)	4.7E − 04	0.037 (0.018)	3.8E − 02	–	**Height** (↓), **trunk/body fat-free mass** (↓), **basal metabolic rate** (↓), **weight** (↓)	+++	+	+	+	++	–
TXNDC15	*TXNDC15*	rs3733897	G	0.38	1.045 (0.025)	2.0E − 317	0.012 (0.004)	4.5E − 04	0.003 (0.015)	0.859	√	**Height** (↓)	++	++	+	++	++	–
PRDX6	*PRDX6*	rs61826753	A	0.33	0.37 (0.025)	1.8E − 51	0.036 (0.011)	6.8E − 04	0.084 (0.015)	5.4E − 08	–	–	+++	+++	+++	+++	+++	–

*2SMR* two sample Mendelian randomization; *EA* effect allele; *EAF* effect allele frequency; *KO* knockout

^a^EA referred to the allele associated with increased protein levels in CKB, and EAF was obtained from Biobank Japan

^b^2SMR: cis-pQTL obtained from CKB, with lookups in Biobank Japan for GWAS summary statistics

^c^Beta and SE were calculated using Wald ratio method

^d^Observation results in CKB in direction of protein-to-BMI

^e^2SMR: cis-pQTL obtained from UKB, with lookups in GIANT for GWAS summary statistics

^f^Traits or diseases in bold: P < 5 × 10^–8^, others were *P* < 5 × 10^–6^

^g^Levels of expression was estimated using GTEx and categorized into three groups as denoted: + (low); ++ (moderate) and +++ (high)

^h^Significant in both directions (protein-to-BMI and BMI-to-protein)

In PheWAS analyses of the eight proteins, *cis*-pQTLs for 6 proteins (ITIH3, LRP11, SCAMP3, NUDT5, OGN, and EFEMP1) were associated, based on PhenoScanner, with several adiposity traits, including BMI, WC and body composition. TXNDC15 *cis*-pQTL was associated with height, while there were no previously reported associations of PRDX6 *cis*-pQTLs with any traits and disease outcomes. The PheWAS analyses using different leading *cis*-pQTLs in UKB for eight proteins found similar results (eTable 11). Moreover, these eight proteins were not strongly correlated with proteins with established associations with regulation of appetite or satiety (r < 0.24), including AGRP, GHRL, NPY, and PYY (eFigure 10). In PheWAS analyses involving GWAS Catalog, we did not find additional adiposity-related traits associated with these eight proteins.

In tissue-specific expression analyses, three proteins (OGN, EFEMP1, PRDX6) were highly expressed in adipose tissues, one (ITIH3) was predominantly expressed in the liver, while the remaining four proteins (LRP11, SCAMP3, NUDT5, TXNDC15) were moderately expressed in multiple tissues (eFigure 11). Further searches of DrugBank, OpenTargets and other databases identified no evidence of drug targets or drug development for all eight proteins.

## Discussion

This study systematically examined the associations of adiposity with a large number of proteins in Chinese adults. Despite the population being relatively lean, adiposity was significantly associated with > 1000 out of ~ 1500 proteins studied. Moreover, genetic analyses provided support for causal and apparently linear effects of BMI on > 300 proteins, especially leptin, FABP4, GOLM2, PON3, and NCAN, with somewhat fewer significant associations but greater effect sizes for the overlapping proteins in men than women. These observational and genetic findings were largely replicated in Europeans with different mean levels of BMI. Enrichment analyses of selected proteins demonstrated that adiposity influenced multiple proteins involved in pathways related to atherosclerosis, lipid metabolism, tumour progression, inflammation and immune function. MR analyses using *cis*-pQTLs identified in CKB GWAS found eight proteins significantly affect levels of BMI, with one protein also showing a bi-directional causal relationship.

In recent decades, several studies have explored the associations of adiposity with plasma levels of proteins, with varying number of proteins measured by different platforms [12–17]. In a combined analysis of 921 SomaScan proteins in 4600 participants from three different populations (Germany, UK, and Qatar), 152 and 24 proteins were significantly associated with BMI in observational and genetic analyses respectively, with leptin, IGFBP1 and IGFBP2 being the strongest hits [17]. A recent UK study measured 3622 proteins using SomaScan platform in 2737 participants and demonstrated that self-reported BMI (mean 25.9 kg/m^2^) was significantly associated with 1576 (44%) proteins [15]. In genetic analyses, however, only eight proteins (0.5%, 8/1576) were significantly associated with BMI, including leptin, FABP4, PILRA and INHBB, compared with 6.9% (55/798) in the present study when applying the same Bonferroni-corrected threshold. The reasons for the discrepant results may reflect differences in the assay platforms used, type of proteins included, the reliability of BMI measured (self-reported vs measured in CKB), or differences in the statistical power of the genetic instruments used (BMI variance explained: 2.8% vs 3.7% in CKB). Nevertheless, the present genetic analyses among relatively lean Chinese adults confirmed associations for three of the four protein hits in that study (leptin, FABPA and PILRA) that were also included in the present OLINK assay platforms.

Recently, the same OLINK platform was used to quantify 1463 proteins in ~ 50,000 UKB participants [16]. In separate analyses of UKB data using the similar adjustment for covariates, we found 1379 and 935 proteins were significantly associated with BMI in observational and genetic analyses, respectively, which replicated > 90% of the BMI-associated proteins identified in CKB. Moreover, there was a high correlation between the effect sizes of the overlapping proteins in both conventional and genetic analyse despite the two populations having different ranges of BMI distribution (mean BMI: 27.4 kg/m^2^ in UKB vs. 23.9 kg/m^2^ in CKB). As for proteins with inconsistent findings, the likely reasons could only be speculated, which may include study power, difference in genetic architecture of specific proteins, and possibility of ancestry-specific mechanisms. Hence, the present study involving Chinese and UK populations provide robust new evidence of causal associations of BMI with plasma levels of a large number of proteins throughout a broad ranges of BMI distribution, which further highlight the generalizability and global relevance of the main study findings.

The enrichment analysis of the top 55 proteins in CKB demonstrated the relevance of these differentially expressed proteins with multiple biological processes, including macrophage-related foam cell differentiation, IL6 production and immune cell function. Indeed, macrophages play a key role in the development of atherosclerotic plaques [32]. Moreover, metabolic processes associated with lipid metabolism were also enriched, which were also relevant to the development and progression of cardiometabolic diseases. In addition, IL-6 production was also enriched highlighting the role of a pro-inflammatory state. Inflammation has been implicated in multiple diseases [33], and adiposity may affect immune system through an enhanced inflammatory state [34]. Obesity can also impair immune function and leucocyte counts. Moreover, obesity also enhances the positive feedback loop between local inflammation in adipose tissue and altered immune response, both contributing to the development and sequelae of cardiometabolic diseases [34]. In analyses using KEGG methods, several adiposity-affected proteins (e.g. CSF1, PGF) were associated with pathways of ECM-receptor interaction and Rap1 signalling. Notably, Rap1 is a crucial player in tumour progression and targeting Rap1 signalling and its regulators could potentially control carcinogenesis, metastasis, chemo-resistance and immune evasion [35]. ECM-receptor interaction signal pathway was also identified as possibly involved in the development of breast cancer [36]. All together, these enrichment findings among relatively lean Chinese adults identified multiple complex pathways by which adiposity may increase disease risks.

The present two-sample bi-directional MR analyses provided causal support for eight proteins that significantly affect the levels of BMI (i.e., with associations in the protein-to-BMI direction). Separate colocalisation analyses showed no strong evidence of their shared causal genetic variants with BMI, which may be attributed partly to the limited study power. Of these eight proteins, three (ITIH3, OGN, TXNDC15) were further replicated using the different *cis*-pQTL identified in the UKB. Two of these three proteins are highly expressed in adipose tissues (OGN) or liver (ITIH3), which could be prioritised as potential drug targets for treating obesity and obesity-related diseases. OGN, also known as mimecan, is secreted extracellularly [37, 38] and is a downstream mediator of NPY signalling (one of the most potent appetite stimulant peptides found in brain) via osteoblastic Y1 receptors, and studies in obese participants have linked OGN with BMI, weight and plasma glucose levels [38]. Thus, the OGN pathway is an attractive target for potential novel treatment of obesity and type 2 diabetes. ITIH3 may act as a carrier of hyaluronic acid in plasma and has been linked with obesity and MI [39]. Moreover, the inverse association of ITIH3 with obesity was also reported in experimental mouse models [40], and in participants with sustained weight loss following caloric restriction diets or bariatric surgery [41]. Combined with relevant experimental evidence, the findings of this study provide support for ITIH3 as a novel potential target for treatment of obesity.

The chief strengths of the present study include assessment of large numbers of proteins, independent replication of the main results in different ancestry populations, use of robust trans-ancestry adiposity genetic instruments, application of bi-directional MR methods, in addition to enrichment analyses to clarify multiple biological processes. Furthermore, the present study included mean levels and ranges of adiposity that differed importantly from those in Western populations. However, the present study also has limitations. First, the study did not consider several other adiposity traits (e.g. WC, WHR, body fat percentage), nor properly investigate proteins showing quadratic associations with adiposity. Second, we could not clarify if any apparent differences between men and women in the genetic analyses were driven by sex-related biological mechanisms or merely an artefact resulting from limited statistical power. Third, our two-sample bi-directional MR only involved a very small number of proteins due to lack of overlapping *cis*-pQTLs in publically-available GWAS summary statistics [42, 43]. Consequently, we were unable to confirm (or refute) previous findings of certain proteins (e.g. LEP, AGER, DPT, and CTSA) that may affect BMI [17]. Fourth, for the main bi-directional MR analyses using the publically available summary genetic data, it would not be possible to fully account for potential collider bias resulting from sample overlapping, although we undertook sensitivity analyses in smaller dataset with much reduced study power to minimise such biases. Future studies involving a larger sample size and better genetic instruments, involving perhaps both *cis*- and *trans*-pQTLs, are needed to further replicate and clarify the effects of different proteins on BMI and other adiposity traits (or vice versa), including protein–protein interactions and evidence of shared causal genetic variants, in different populations.

Overall, this study of relatively lean Chinese adults demonstrated that adiposity was significantly associated with a large number of proteins, with support for the causal relevance of > 300 proteins in the BMI-to-protein direction. Bi-directional MR analyses also found eight proteins may affect levels of adiposity, which may inform future drug development. Combined with enrichment analyses and available experimental data, the present study identified multiple pathways by which adiposity may increase disease risks and provide support for novel protein targets for potential treatment of obesity and obesity-related diseases.

### Supplementary Information

Below is the link to the electronic supplementary material.

Supplementary file1 (DOCX 2876 KB)

Supplementary file2 (XLSX 357 KB)

## Data Availability

The China Kadoorie Biobank (CKB) is a global resource for the investigation of lifestyle, environmental, blood biochemical and genetic factors as determinants of common diseases. The CKB study group is committed to making the cohort data available to the scientific community in China, the UK and worldwide to advance knowledge about the causes, prevention and treatment of disease. For detailed information on what data is currently available to open access users and how to apply for it, please visit: http://www.ckbiobank.org/site/Data+Access. A research proposal will be requested to ensure that any analysis is performed by *bona fide* researchers. Researchers who are interested in obtaining additional information or data that underlines this paper should contact ckbaccess@ndph.ox.ac.uk. For any data that are not currently available for open access, researchers may need to develop formal collaboration with study group.

## Abbreviations

ADAMTS15: A disintegrin and metalloproteinase with thrombospondin motifs 15
AGER: Advanced glycosylation end product-specific receptor
AGRP: Agouti-related protein
BMI: Body mass index
BBJ: Biobank Japan
CA14: Carbonic anhydrase 14
CD276: Cluster of Differentiation 276
CDHR2: Cadherin-related family member 2
CEACAM5: Carcinoembryonic antigen-related cell adhesion molecule 5
CHGB: Secretogranin-1
CI: Confidence interval
*cis*-pQTL: *cis*-Protein quantitative trait locus
CKB: China Kadoorie Biobank
CKMT1A_CKMT1B: Creatine kinase U-type, mitochondrial
CPM: Carboxypeptidase M
CTSA: Lysosomal protective protein
CXCL17: C-X-C motif chemokine 17
DPT: Dermatopontin
EFEMP1: EGF-containing fibulin-like extracellular matrix protein 1
FABP4: Fatty acid-binding protein, adipocyte
FDR: False discovery rate
FGFBP1: Fibroblast growth factor-binding protein 1
GHRL: Appetite-regulating hormone
GIANT: Genetic Investigation of ANthropometric Traits
GO: Gene ontology
GOLM2: Protein GOLM2
GS: Genetic score
GTEX: Genotype-Tissue Expression
GWAS: Genome-wide association study
IGFBP1: Insulin-like growth factor-binding protein 1
IGFBP2: Insulin-like growth factor-binding protein 2
IHD: Ischaemic heart disease
IL1RN: Interleukin-1 receptor antagonist protein
ITIH3: Inter-alpha-trypsin inhibitor heavy chain H3
IL6: Interleukin-6
IL8: Interleukin-8
INHBB: Inhibin beta B chain
LAYN: Layilin
LEPR: Leptin receptor
LSP1: Lymphocyte-specific protein 1
LOD: Limit of detection
LRP11: Low-density lipoprotein receptor-related protein 11
MGI: Mouse Genome Informatics
MOG: Myelin oligodendrocyte glycoprotein
MR: Mendelian randomisation
MSLN: Mesothelin
NCAM2: Neural cell adhesion molecule 2
NCAN: Neurocan core protein
NECTIN4: Nectin-4
NFASC: Neurofascin
NPX: Normalized Protein eXpression
NPY: Pro-neuropeptide Y
NUDT5: ADP-sugar pyrophosphatase
MMP12: Matrix Metallopeptidase 12
OGN: Mimecan
OXT: Oxytocin-neurophysin 1
PILRA: Paired immunoglobulin-like type 2 receptor alpha
PON3: Serum paraoxonase/lactonase 3
PRDX6: Peroxiredoxin-6
PYY: Peptide YY
QC: Quality control
SCAMP3: Secretory carrier-associated membrane protein 3
SD: Standard deviation
SLAMF7: SLAM family member 7
SSC4D: Scavenger receptor cysteine-rich domain-containing group B protein
TNF: Tumor necrosis factor
TNFRSF6B: Tumor necrosis factor receptor superfamily, member 6b
TXNDC15: Thioredoxin domain-containing protein 15
UKB: UK Biobank
VCAM1: Vascular cell adhesion protein 1
WC: Waist circumference
WHR: Waist hip ratio

## Code availability

Custom code was used all statistical analyses in this report.
