# Supplementary material for: Conventional and genetic associations of adiposity with 1463 proteins in relatively lean Chinese adults
Source: Eur J Epidemiol. Author manuscript; Available in PMC 2023 Oct 18. (PMC10570181; doi:10.1007/s10654-023-01038-9)

Conventional and genetic associations of adiposity with 1463 proteins in relatively lean Chinese adults

Supplementary Material

Contents

Members of the China Kadoorie Biobank collaborative group 2
eTable 1. List of 1463 proteomics quantified by the OLINK Explore I assay 3
eTable 2. Number of proteins with QC warnings 4
eTable 3. Associations of potential confounders with BMI GS 5
eTable 4. Baseline characteristics of participants 6
eTable 5. Difference of 1463 proteins associated with 1-SD higher BMI in conventional analyses 7
eTable 6. Number of proteins significantly associated at FDR<0.05 with BMI in conventional analyses, with additional adjustments or exclusions 8
eTable 7. Difference of 1463 proteins associated with 1-SD higher BMI in genetic analyses 9
eTable 8. List of significantly enriched biological process terms of BMI-related proteins 10
eTable 9. List of significantly enriched biological process terms of 1463 OLINK proteins 11
eTable 10. List of significantly enriched KEGG pathways of BMI-related proteins 12
eTable 11. PheWAS associations of *cis*-pQTL in UKB of eight proteins showing causal relationship with BMI 13
eFigure 1. Flow diagram of study design and participant selection in CKB 14
eFigure 2. Distributions of individual proteins (n=1463), by OLINK panel 15
eFigure 3. Volcano plots of associations of 1463 proteins with 1-SD higher BMI in observational analyses, by OLINK panel 16
eFigure 4. Conventional linear associations of BMI with 20 selected proteins, by OLINK panel 17
eFigure 5. Volcano plots of associations of 1463 proteins with 1-SD higher BMI in conventional analyses of subcohort participants, by OLINK panel 18
eFigure 6. Global comparisons of associations of 1463 proteins with 1-SD higher BMI in observational and genetic analyses, between men and women 19
eFigure 7. Genetic non-linear associations of BMI with 20 selected proteins, by OLINK panel 20
eFigure 8. Volcano plots of associations of 1463 proteins with 1-SD higher BMI in genetic analyses, by OLINK panel 21
eFigure 9. Volcano plot of associations of 1463 proteins with 1-SD higher BMI in genetic analyses of subcohort participants, by OLINK panel 22
eFigure 10. Correlation matrix among those adiposity-associated proteins in CKB 23
eFigure 11. Human tissue-specific gene expression for the eight proteins showing causal relationship with adiposity 24

eTable 1. List of 1463 proteomics quantified by the OLINK Explore I assay

Saved into the Excel file as supplementary material.

eTable 2. Number of proteins with QC warnings

Number of QC warnings per protein, n (%)[1]	Number of proteins, n (%)[2]
9 (0.2%)	108 (7.3%)
20 (0.5%)	62 (4.2%)
23 (0.6%)	63 (4.3%)
25 (0.6%)	115 (7.8%)
31 (0.8%)	86 (5.8%)
32 (0.8%)	76 (5.2%)
34 (0.9%)	62 (4.2%)
45 (1.1%)	94 (6.4%)
53 (1.3%)	110 (7.5%)
58 (1.5%)	114 (7.7%)
90 (2.3%)	110 (7.5%)
115 (2.9%)	105 (7.1%)
139 (3.5%)	80 (5.4%)
141 (3.5%)	71 (4.8%)
148 (3.7%)	110 (7.5%)
158 (4.0%)	106 (7.2%)

[1] A total of 3977 samples
[2] A total of 1463proteins

eTable 3. Associations of potential confounders with BMI GS

Confounder	OR or β (95% CI) per 1-SD higher genetically-instrumented BMI
Ever-regular smoker	1.08 (0.97, 1.20)
Ever-regular alcohol drinker	0.96 (0.86, 1.06)
Physical activity	0.94 (0.85, 1.03)
Education	1.01 (0.92, 1.10)
Income	1.02 (0.95, 1.10)
Standing height, cm	0.00 (-0.17, 0.17)
RPG, mmol/L	0.21 (0.11, 0.31)
SBP, mmHg	1.23 (0.55, 1.92)
Waist, cm	1.57 (1.28, 1.86)

The model was adjusted for age at baseline, age squared, sex, 10 regions, and 12 PCs. Potential confounders were dichotomised: ever-regular smoker (yes vs no), ever-regular alcohol drinker (yes vs no), total PA (≥30 vs <30 MET-h/day), education (≥9 vs <9 years), and household income (≥10,000 vs <10,000 RMB/year). The analyses of smoking and alcohol were conducted in men due to the small number of women who smoked (4%) or drank (2%).

eTable 4. Baseline characteristics of participants

Characteristics [a]	All (n=3977)	Subcohort (n=2026)	IHD Cases (n=1951)
**Age and socioeconomic factors**			
Age, years (SD)	57.3 (11.6)	51.3 (10.4)	52.2 (10.6)
Women, %	53.7	62.1	45.1
Urban, %	48.8	50.6	47.0
≥6 years of education, %	45.1	52.1	37.7
**Anthropometry and blood pressure, mean (SD)**			
BMI, kg/m^2	23.9 (3.3)	23.9 (3.2)	24.0 (3.4)
Waist circumference, cm	81.9 (9.1)	80.3 (8.7)	83.6 (9.4)
SBP, mmHg	138.3 (22.0)	130.6 (19.7)	146.3 (23.5)
Fasting time	4.7 (4.1)	5.1 (4.3)	4.3 (4.0)
**Lifestyle factors**			
Ever regular smoker**, %**			
Men	75.0	74.9	75.1
Women	5.8	3.3	9.3
Regular alcohol consumption**, %**			
Men	34.6	40.1	30.6
Women	3.0	2.7	3.4
Physical activity, MET-h/day (SD)	17.3 (10.7)	21.3 (12.3)	13.0 (8.7)
**Medical history and health status,[b] %**			
Self-rated poor health	16.6	13.4	19.8
Diabetes	11.2	16.1	6.4
Chronic kidney disease	1.4	1.4	1.3
Cancer	0.6	0.6	0.6

[a] Adjusted for age, sex and study area, as appropriate.
[b] Based on self-report, while for diabetes, those with screen-detected cases at baseline were also included.
Abbreviations: SD=standard deviation; BMI= body mass index; SBP=systolic blood pressure; MET= metabolic equivalent of task.

eTable 5. Difference of 1463 proteins associated with 1-SD higher BMI in conventional analyses

Saved into the Excel file as supplementary material

eTable 6. Number of proteins significantly associated at FDR<0.05 with BMI in conventional analyses, with additional adjustments or exclusions

	Cardiometabolic (n=369)	Inflammation (n=368)	Neurology (n=367)	Oncology (n=368)	Total (n=1463)*
Main results	288	259	266	289	1096
Adjustment					
Diabetes	284	264	260	284	1086
Kidney disease	284	264	261	284	1087
Cancer	284	264	262	285	1089
Exclusion					
Diabetes	280	255	259	277	1065
Kidney disease	278	253	254	279	1059
Cancer	280	253	255	280	1063
QC warning	289	262	265	290	1100

* Three proteins (IL6, CXCL8 and TNF) were included in all four panels, resulting in a total of 1463 unique proteins.

eTable 7. Difference of 1463 proteins associated with 1-SD higher BMI in genetic analyses

Saved into an Excel file as supplementary material

eTable 8. List of significantly enriched biological process terms of BMI-related proteins

Saved into an Excel file as supplementary material

eTable 9. List of significantly enriched biological process terms of 1463 OLINK proteins

Saved into an Excel file as supplementary material

eTable 10. List of significantly enriched KEGG pathways of BMI-related proteins

Saved into an Excel file as supplementary material

eTable 11. PheWAS associations of *cis*-pQTL in UKB of eight proteins showing causal relationship with BMI

Protein	rsID	PheWAS associations [a]
ITIH3	rs9881468	**BMI, height, impedance of leg/arm/whole body**
LRP11	rs2342858	hand grip strength, BMI
SCAMP3	rs1142287	BMI
NUDT5	rs10508438	BMI
OGN	rs7026361	**height, impedance of arm/body**
EFEMP1	rs59985551	**height, trunk/body fat-free mass**
TXNDC15	rs3733897	**height**
PRDX6	rs33951697	-

a Traits or diseases in bold: P < 5*10-8 , others were P < 5*10-6

eFigure 1. Flow diagram of study design and participant selection in CKB

*Selection for the subcohort used simple random sampling; †Individuals may be included in more than one study arm

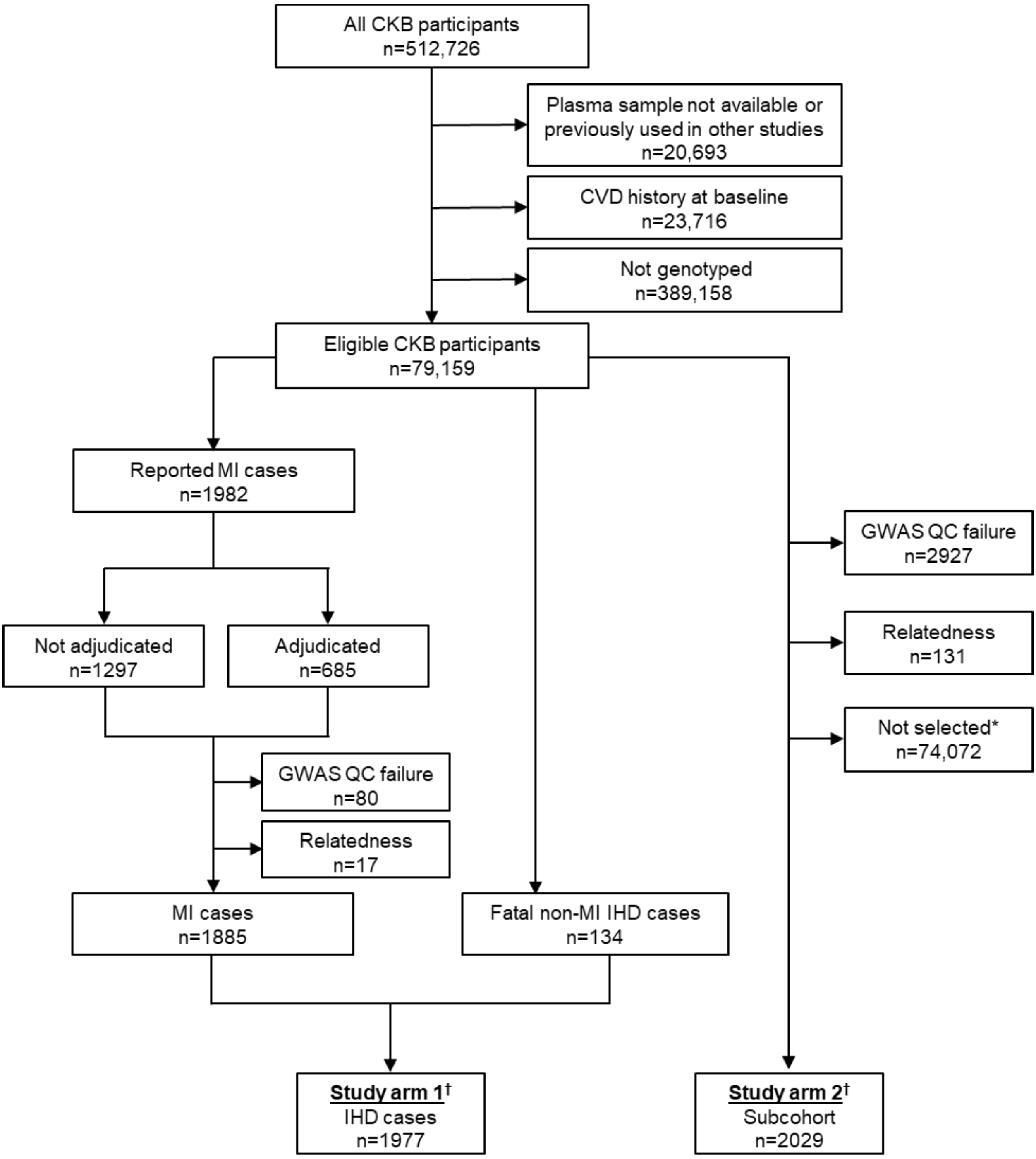

eFigure 2. Distributions of individual proteins (n=1463), by OLINK panel

Separate pdf files

eFigure 3. Volcano plots of associations of 1463 proteins with 1-SD higher BMI in observational analyses, by OLINK panel

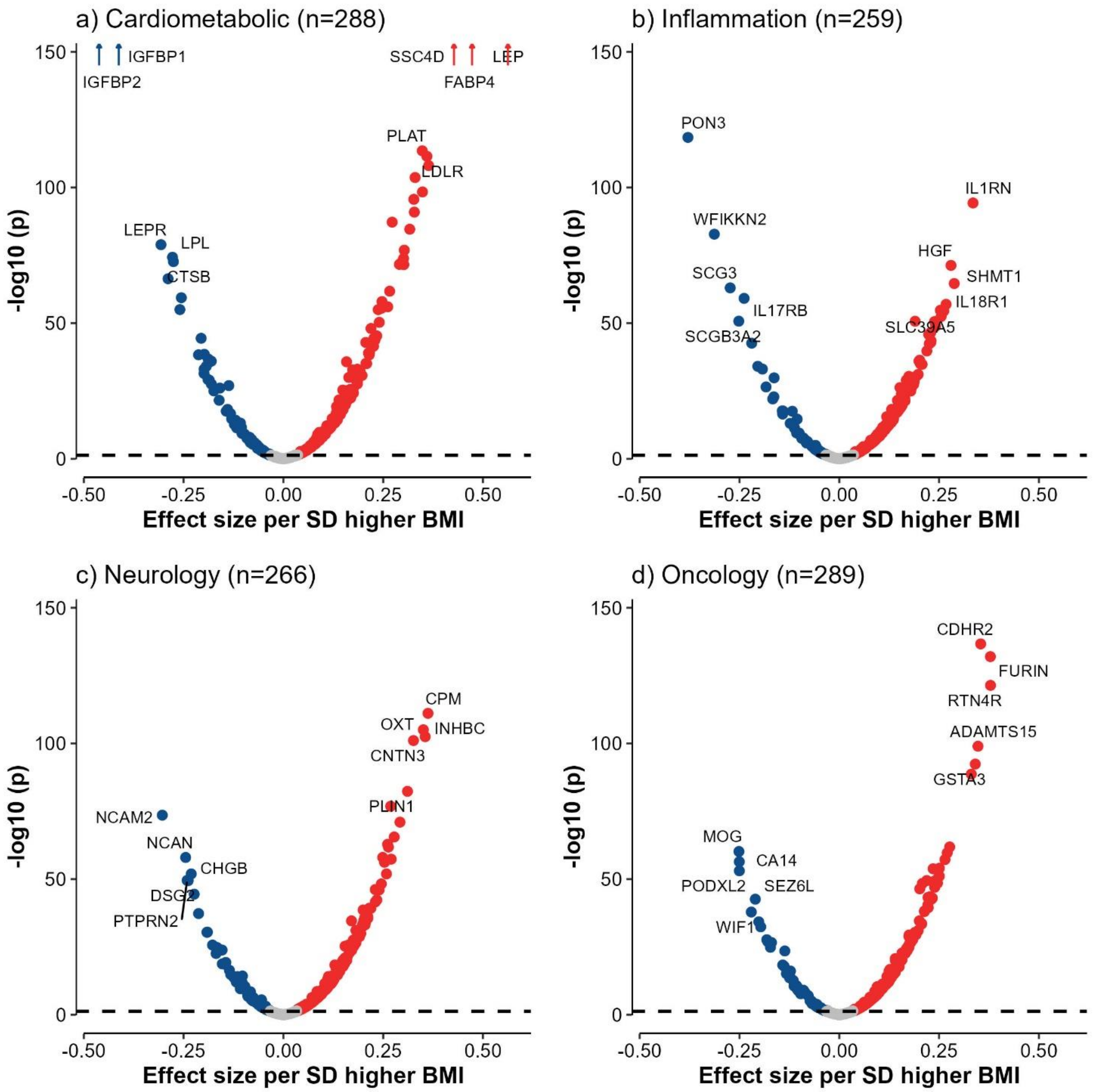

eFigure 4. Conventional linear associations of BMI with 20 selected proteins, by OLINK panel

To examine the shape of the associations in observation analyses, adjusted means of proteins were calculated within each of BMI quintiles using multiple linear regression and then plotted against mean BMI within each of quintiles. Within each panel, top 5 proteins were selected including 4 positive and 1 inverse proteins.The length of y-axis represents ± 2 SD from the mean of the corresponding protein.

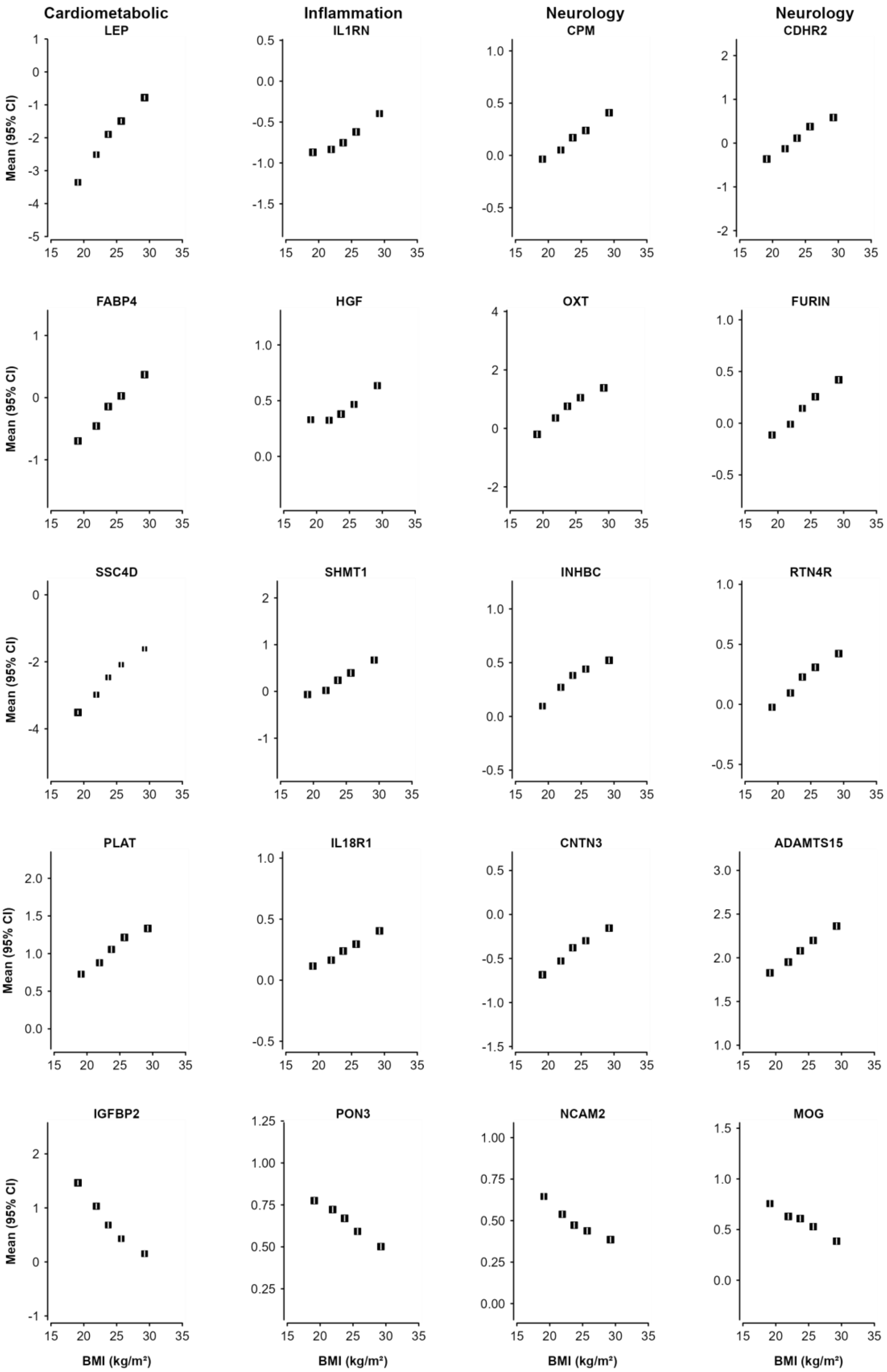

eFigure 5. Volcano plots of associations of 1463 proteins with 1-SD higher BMI in conventional analyses of subcohort participants, by OLINK panel

Conventional epidemiological analyses for BMI. Results are adjusted for age, age square, sex, study area, fasting time, ambient temperature and plate ID.

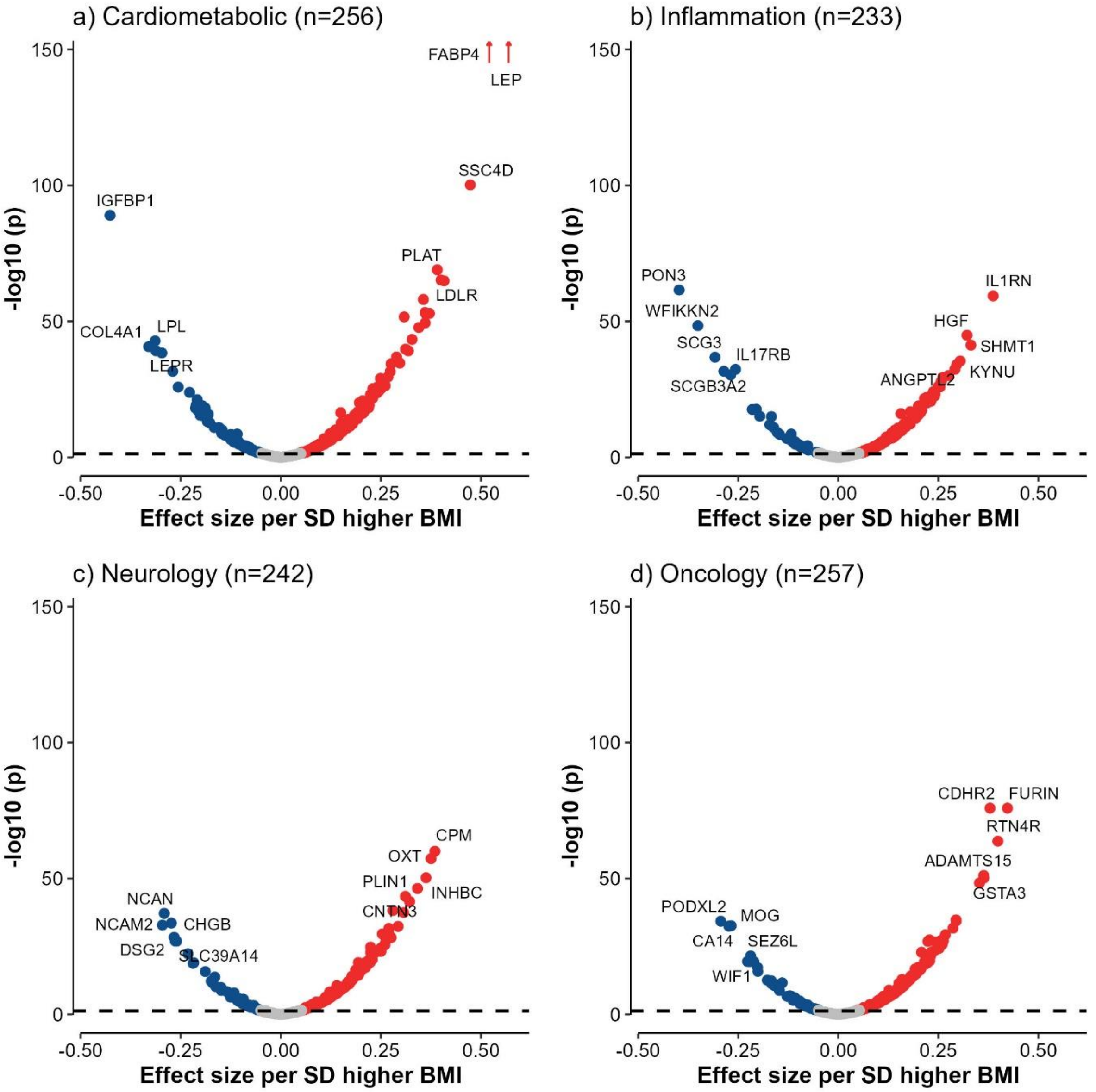

eFigure 6. Global comparisons of associations of 1463 proteins with 1-SD higher BMI in observational and genetic analyses, between men and women

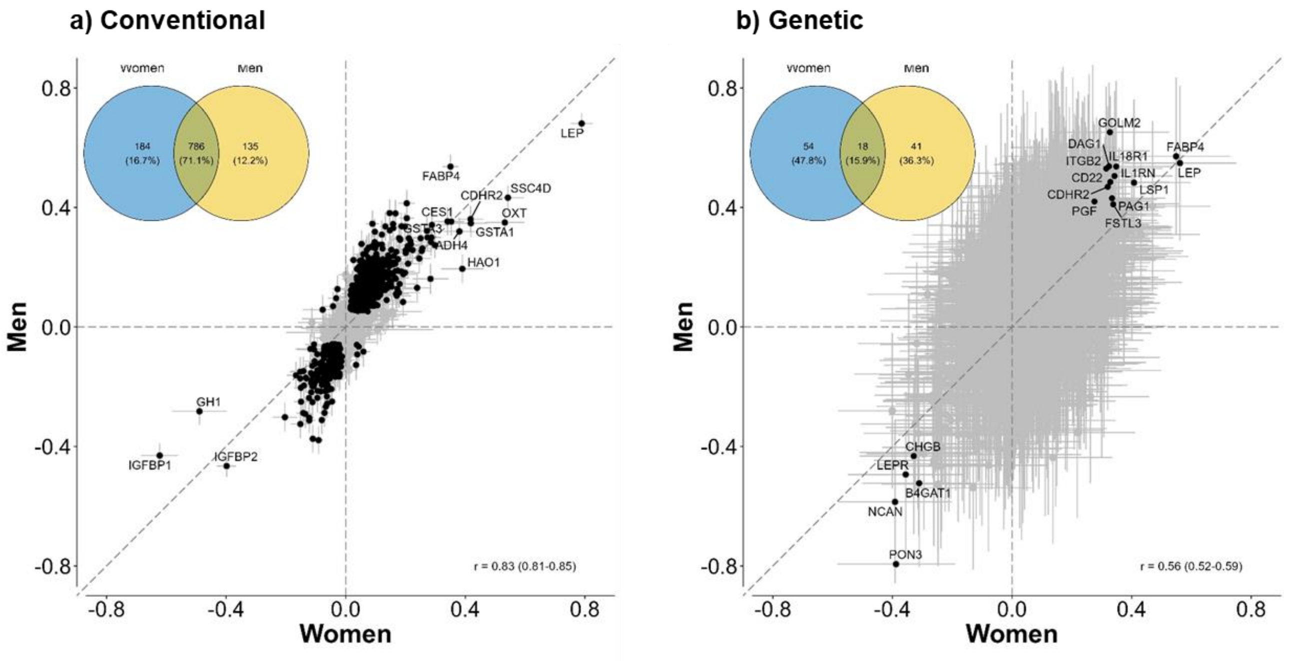

eFigure 7. Genetic non-linear associations of BMI with 20 selected proteins, by OLINK panel

Within each panel, top 5 proteins were selected including 4 positive and 1 inverse proteins. Piecewise linear method was used to calculate the estimates (adjusted for age, age squared, sex, and study area [ten groups], ascertainment, and 12 national PCs). Each line segment begins where the previous segment finished (black lines) and the intercept was set to the population mean BMI (red dot). The 95% CI in the genetic analyses are represented by the shaded patterns. The length of y-axis represents approximately ± 2 SD from the mean of the corresponding protein.

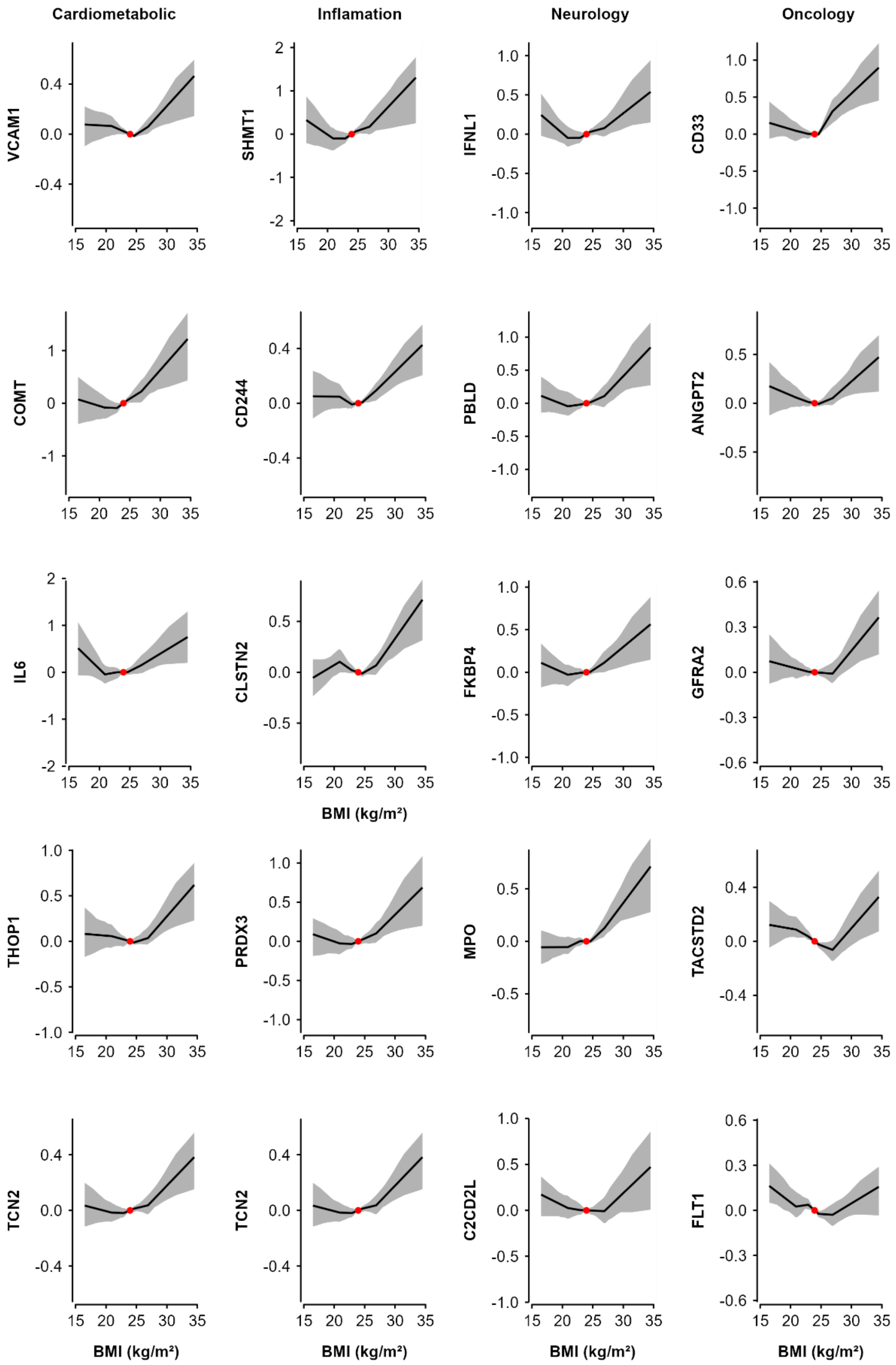

eFigure 8. Volcano plots of associations of 1463 proteins with 1-SD higher BMI in genetic analyses, by OLINK panel

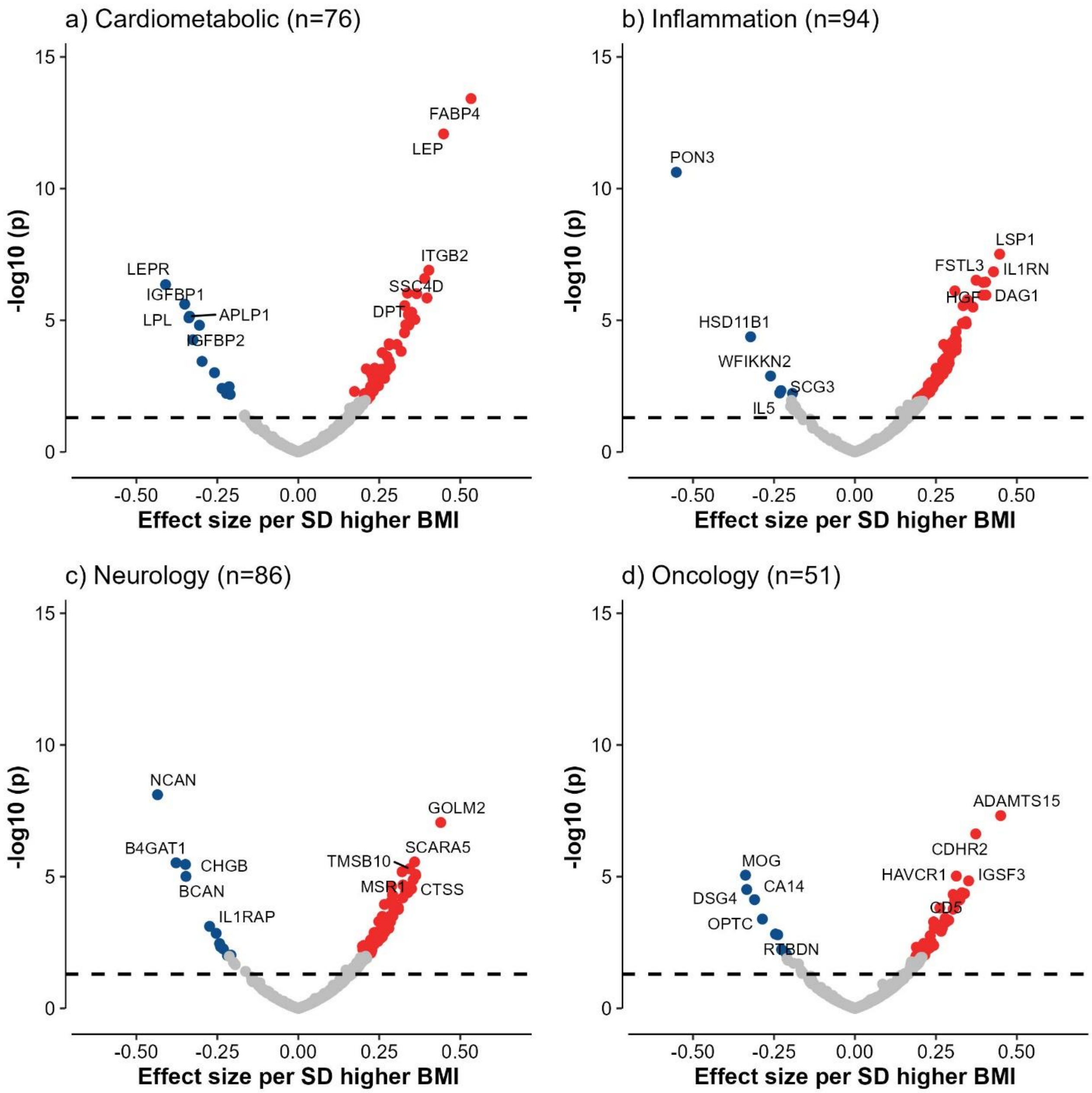

eFigure 9. Volcano plot of associations of 1463 proteins with 1-SD higher BMI in genetic analyses of subcohort participants, by OLINK panel

Genetic epidemiological analyses for BMI. Results are adjusted for age, age square, sex, study area, fasting time, ambient temperature, and the first 12 PCs.

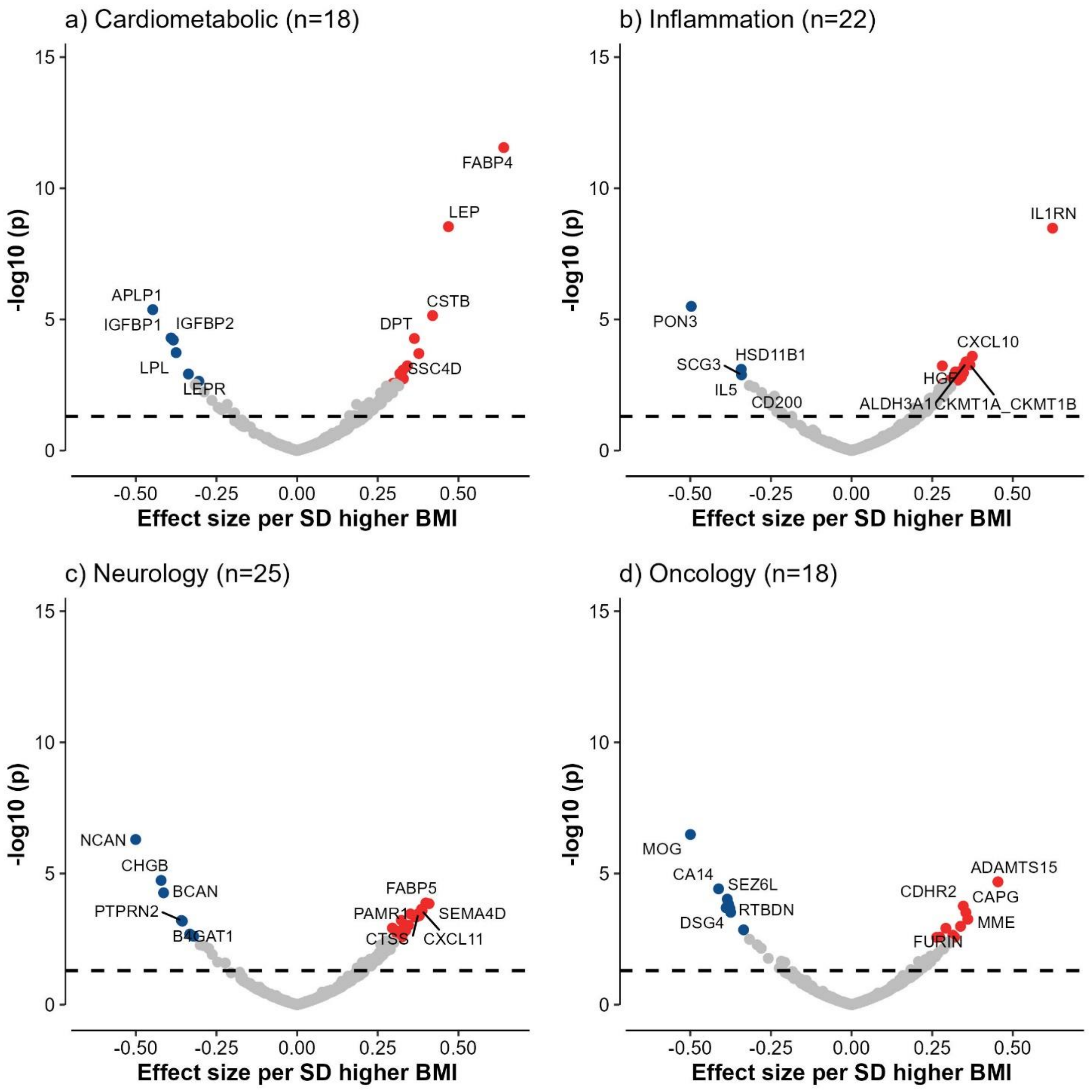

eFigure 10. Correlation matrix among those adiposity-associated proteins in CKB

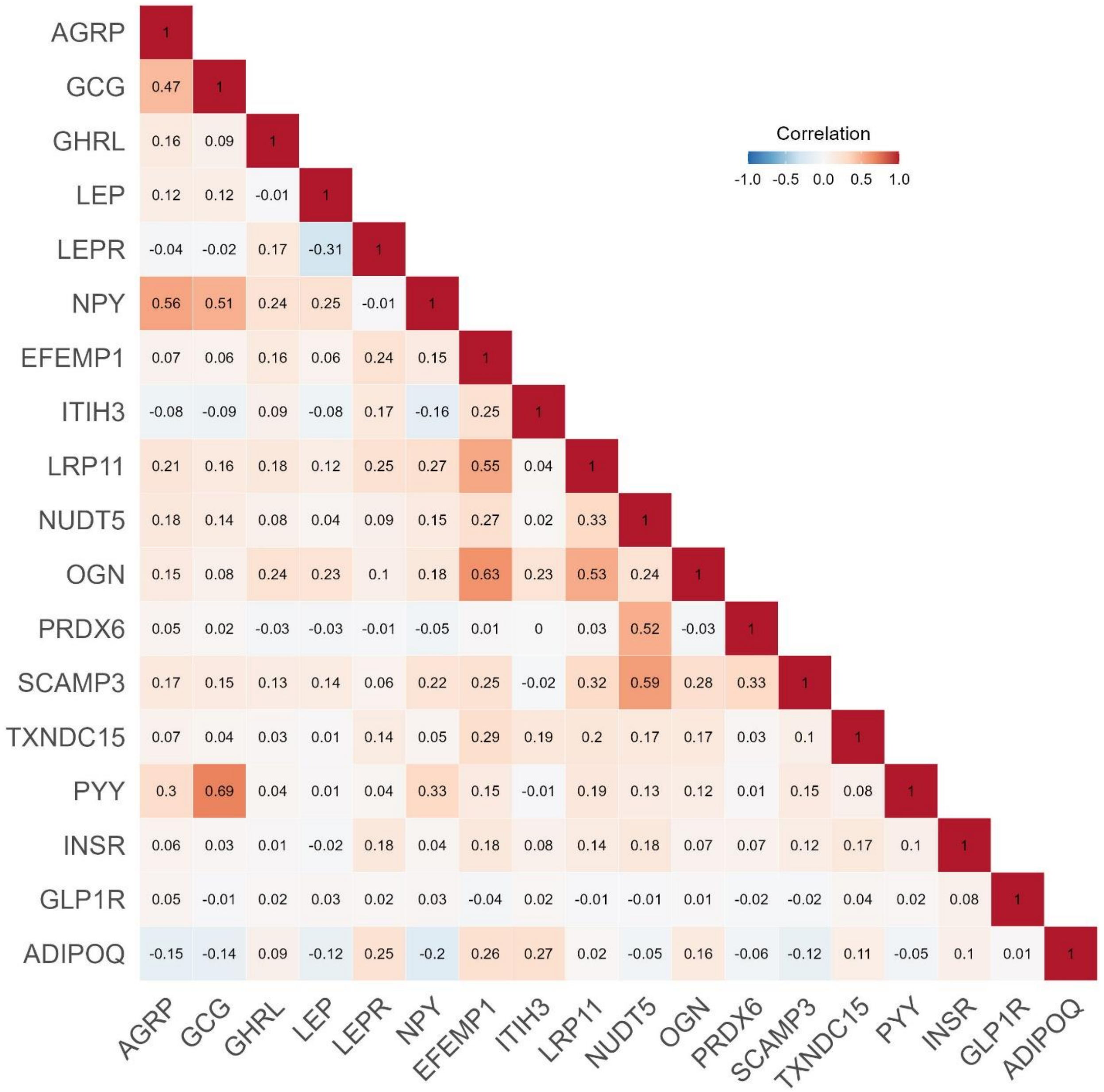

eFigure 11. Human tissue-specific gene expression for the eight proteins showing causal relationship with adiposity

Human RNA-seq data from GTEx showing the transcript per million (TPM) expression values for the genes encoding the proteins

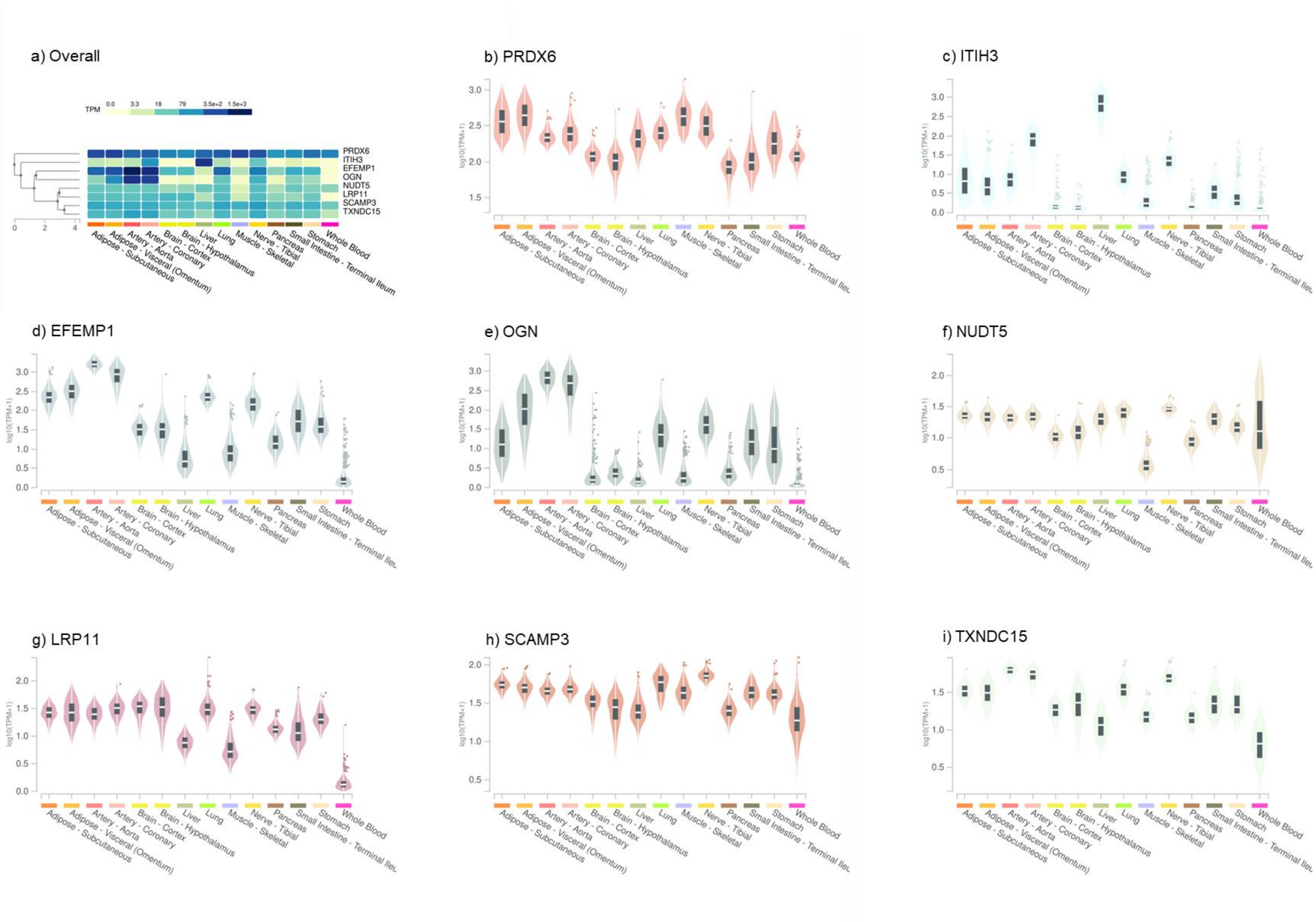

Supplement: Supplementary Material [file EMS187675-supplement-Supplementary_Material.pdf]
