# Supplementary material for: Conventional and genetic associations of adiposity with 1463 proteins in relatively lean Chinese adults
Source: Eur J Epidemiol. 2023 Sep 7;38(10):1089–103. doi: 10.1007/s10654-023-01038-9 (PMC10570181; doi:10.1007/s10654-023-01038-9)
Supplement: Supplementary file 1 [file 10654_2023_1038_MOESM1_ESM.docx]

**Conventional and genetic associations of adiposity with 1463 proteins in relatively lean Chinese adults**

**Supplementary Material**

**Contents**

Members of the China Kadoorie Biobank collaborative group	2

eTable 1. List of 1463 proteomics quantified by the OLINK Explore I assay	3

eTable 2. Number of proteins with QC warnings	4

eTable 3. Associations of potential confounders with BMI GS	5

eTable 4. Baseline characteristics of participants	6

eTable 5. Difference of 1463 proteins associated with 1-SD higher BMI in conventional analyses	7

eTable 6. Number of proteins significantly associated at FDR<0.05 with BMI in conventional analyses, with additional adjustments or exclusions	8

eTable 7. Difference of 1463 proteins associated with 1-SD higher BMI in genetic analyses	9

eTable 8. List of significantly enriched biological process terms of BMI-related proteins	10

eTable 9. List of significantly enriched biological process terms of 1463 OLINK proteins	11

eTable 10. List of significantly enriched KEGG pathways of BMI-related proteins	12

eTable 11. PheWAS associations of *cis*-pQTL in UKB of eight proteins showing causal relationship with BMI	13

eFigure 1. Flow diagram of study design and participant selection in CKB	14

eFigure 2. Distributions of individual proteins (n=1463), by OLINK panel	15

eFigure 3. Volcano plots of associations of 1463 proteins with 1-SD higher BMI in observational analyses, by OLINK panel	16

eFigure 4. Conventional linear associations of BMI with 20 selected proteins, by OLINK panel	17

eFigure 5. Volcano plots of associations of 1463 proteins with 1-SD higher BMI in conventional analyses of subcohort participants, by OLINK panel	18

eFigure 6. Global comparisons of associations of 1463 proteins with 1-SD higher BMI in observational and genetic analyses, between men and women	19

eFigure 7. Genetic non-linear associations of BMI with 20 selected proteins, by OLINK panel	20

eFigure 8. Volcano plots of associations of 1463 proteins with 1-SD higher BMI in genetic analyses, by OLINK panel	21

eFigure 9. Volcano plot of associations of 1463 proteins with 1-SD higher BMI in genetic analyses of subcohort participants, by OLINK panel	22

eFigure 10. Correlation matrix among those adiposity-associated proteins in CKB	23

eFigure 11. Human tissue-specific gene expression for the eight proteins showing causal relationship with adiposity	24

### **Members of the China Kadoorie Biobank collaborative group**

**International Steering Committee:** Junshi Chen, Zhengming Chen (PI), Robert Clarke, Rory Collins, Liming Li (PI), Chen Wang, Jun Lv, Richard Peto, Robin Walters.

**International Co-ordinating Centre, Oxford:** Daniel Avery, Derrick Bennett, Ruth Boxall, Sushila Burgess, Ka Hung Chan, Yiping Chen, Zhengming Chen, Johnathan Clarke, Robert Clarke, Huaidong Du, Ahmed Edris Mohamed, Hannah Fry, Simon Gilbert, Pek Kei Im, Andri Iona, Maria Kakkoura, Christiana Kartsonaki, Hubert Lam, Kuang Lin, James Liu, Mohsen Mazidi, Iona Millwood, Sam Morris, Qunhua Nie, Alfred Pozarickij, Paul Ryder, Saredo Said, Dan Schmidt, Becky Stevens, Iain Turnbull, Robin Walters, Baihan Wang, Lin Wang, Neil Wright, Ling Yang, Xiaoming Yang, Pang Yao. 

**National Co-ordinating Centre, Beijing:** Xiao Han, Can Hou, Qingmei Xia, Chao Liu, Jun Lv, Pei Pei, Dianjanyi Sun, Canqing Yu,.

**10 Regional Co-ordinating Centres:** 

**Guangxi** Provincial CDC: Naying Chen, Duo Liu, Zhenzhu Tang. Liuzhou CDC: Ningyu Chen, Qilian Jiang, Jian Lan, Mingqiang Li, Yun Liu, Fanwen Meng, Jinhuai Meng, Rong Pan, Yulu Qin, Ping Wang, Sisi Wang, Liuping Wei, Liyuan Zhou. **Gansu** Provincial CDC: Caixia Dong, Pengfei Ge, Xiaolan Ren. Maiji CDC: Zhongxiao Li, Enke Mao, Tao Wang, Hui Zhang, Xi Zhang. **Hainan** Provincial CDC: Jinyan Chen, Ximin Hu, Xiaohuan Wang. Meilan CDC: Zhendong Guo, Huimei Li, Yilei Li, Min Weng, Shukuan Wu. **Heilongjiang** Provincial CDC:  Shichun Yan, Mingyuan Zou, Xue Zhou. Nangang CDC: Ziyan Guo, Quan Kang, Yanjie Li, Bo Yu, Qinai Xu. **Henan** Provincial CDC: Liang Chang, Lei Fan, Shixian Feng, Ding Zhang, Gang Zhou. Huixian CDC: Yulian Gao, Tianyou He, Pan He, Chen Hu, Huarong Sun, Xukui Zhang. **Hunan** Provincial CDC: Biyun Chen, Zhongxi Fu, Yuelong Huang, Huilin Liu, Qiaohua Xu, Li Yin. Liuyang CDC: Huajun Long, Xin Xu, Hao Zhang, Libo Zhang. **Jiangsu** Provincial CDC: Jian Su, Ran Tao, Ming Wu, Jie Yang, Jinyi Zhou, Yonglin Zhou. Suzhou CDC: Yihe Hu, Yujie Hua, Jianrong Jin, Fang Liu, Jingchao Liu, Yan Lu, Liangcai Ma, Aiyu Tang, Jun Zhang. **Qingdao** CDC: Liang Cheng, Ranran Du, Ruqin Gao, Feifei Li, Shanpeng Li, Yongmei Liu, Feng Ning, Zengchang Pang, Xiaohui Sun, Xiaocao Tian, Shaojie Wang, Yaoming Zhai, Hua Zhang, Licang CDC: Wei Hou, Silu Lv, Junzheng Wang. **Sichuan** Provincial CDC: Xiaofang Chen, Xianping Wu, Ningmei Zhang, Weiwei Zhou. Pengzhou CDC: Xiaofang Chen, Jianguo Li, Jiaqiu Liu, Guojin Luo, Qiang Sun, Xunfu Zhong. **Zhejiang** Provincial CDC: Weiwei Gong, Ruying Hu, Hao Wang,Meng Wang, Min Yu. Tongxiang CDC: Lingli Chen, Qijun Gu, Dongxia Pan，Chunmei Wang, Kaixu Xie, Xiaoyi Zhang. 

### eTable 1. List of 1463 proteomics quantified by the OLINK Explore I assay

Saved into the Excel file as supplementary material. 

| eTable 2. Number of proteins with QC warnings |  |
| --- | --- |
| **Number of QC warnings per protein, n (%)**^**1**^ | **Number of proteins, n (%)**^**2**^ |
| 9 (0.2%) | 108 (7.3%) |
| 20 (0.5%) | 62 (4.2%) |
| 23 (0.6%) | 63 (4.3%) |
| 25 (0.6%) | 115 (7.8%) |
| 31 (0.8%) | 86 (5.8%) |
| 32 (0.8%) | 76 (5.2%) |
| 34 (0.9%) | 62 (4.2%) |
| 45 (1.1%) | 94 (6.4%) |
| 53 (1.3%) | 110 (7.5%) |
| 58 (1.5%) | 114 (7.7%) |
| 90 (2.3%) | 110 (7.5%) |
| 115 (2.9%) | 105 (7.1%) |
| 139 (3.5%) | 80 (5.4%) |
| 141 (3.5%) | 71 (4.8%) |
| 148 (3.7%) | 110 (7.5%) |
| 158 (4.0%) | 106 (7.2%) |
| ^1 ^A total of 3977 samples ^2^ A total of 1463proteins |  |

| eTable 3. Associations of potential confounders with BMI GS |  |
| --- | --- |
| **Confounder** | **OR or** β **(95% CI)** **per 1-SD higher genetically-instrumented BMI** |
| Ever-regular smoker | 1.08 (0.97, 1.20) |
| Ever-regular alcohol drinker | 0.96 (0.86, 1.06) |
| Physical activity | 0.94 (0.85, 1.03) |
| Education | 1.01 (0.92, 1.10) |
| Income | 1.02 (0.95, 1.10) |
| Standing height, cm | 0.00 (-0.17, 0.17) |
| RPG, mmol/L | 0.21 (0.11, 0.31) |
| SBP, mmHg | 1.23 (0.55, 1.92) |
| Waist, cm | 1.57 (1.28, 1.86) |
| The model was adjusted for age at baseline, age squared, sex, 10 regions, and 12 PCs. Potential confounders were dichotomised: ever-regular smoker (yes vs no), ever-regular alcohol drinker (yes vs no), total PA (≥30 vs <30 MET-h/day), education (≥9 vs <9 years), and household income (≥10,000 vs <10,000 RMB/year). The analyses of smoking and alcohol were conducted in men due to the small number of women who smoked (4%) or drank (2%). |  |

| eTable 4. Baseline characteristics of participants |  |  |  |
| --- | --- | --- | --- |
| **Characteristics** ^**a**^ | **All** **(n=3977)** | **Subcohort** **(n=2026)** | **IHD Cases** **(n=1951)** |
| **Age and socioeconomic factors** |  |  |  |
| Age, years (SD) | 57.3 (11.6) | 51.3 (10.4) | 52.2 (10.6) |
| Women, % | 53.7 | 62.1 | 45.1 |
| Urban, % | 48.8 | 50.6 | 47.0 |
| ≥6 years of education, % | 45.1 | 52.1 | 37.7 |
| **Anthropometry and blood pressure, mean (SD)** |  |  |  |
| BMI, kg/m^2^ | 23.9 (3.3) | 23.9 (3.2) | 24.0 (3.4) |
| Waist circumference, cm | 81.9 (9.1) | 80.3 (8.7) | 83.6 (9.4) |
| SBP, mmHg | 138.3 (22.0) | 130.6 (19.7) | 146.3 (23.5) |
| Fasting time | 4.7 (4.1) | 5.1 (4.3) | 4.3 (4.0) |
| **Lifestyle factors** |  |  |  |
| Ever regular smoker**, %** |  |  |  |
| Men | 75.0 | 74.9 | 75.1 |
| Women | 5.8 | 3.3 | 9.3 |
| Regular alcohol consumption**, %** |  |  |  |
| Men | 34.6 | 40.1 | 30.6 |
| Women | 3.0 | 2.7 | 3.4 |
| Physical activity, MET-h/day (SD) | 17.3 (10.7) | 21.3 (12.3) | 13.0 (8.7) |
| **Medical history and health status,**^b^ **%** |  |  |  |
| Self-rated poor health | 16.6 | 13.4 | 19.8 |
| Diabetes | 11.2 | 16.1 | 6.4 |
| Chronic kidney disease | 1.4 | 1.4 | 1.3 |
| Cancer | 0.6 | 0.6 | 0.6 |
| ^a^ Adjusted for age, sex and study area, as appropriate. ^b^ Based on self-report, while for diabetes, those with screen-detected cases at baseline were also included. Abbreviations: SD=standard deviation; BMI= body mass index; SBP=systolic blood pressure; MET= metabolic equivalent of task. |  |  |  |

### eTable 5. Difference of 1463 proteins associated with 1-SD higher BMI in conventional analyses

Saved into the Excel file as supplementary material 

| eTable 6. Number of proteins significantly associated at FDR<0.05 with BMI in conventional analyses, with additional adjustments or exclusions |  |  |  |  |  |
| --- | --- | --- | --- | --- | --- |
|  | **Cardiometabolic** **(n=369)** | **Inflammation** **(n=368)** | **Neurology** **(n=367)** | **Oncology** **(n=368)** | **Total** **(n=1463)*** |
| Main results | 288 | 259 | 266 | 289 | 1096 |
| Adjustment |  |  |  |  |  |
| Diabetes | 284 | 264 | 260 | 284 | 1086 |
| Kidney disease | 284 | 264 | 261 | 284 | 1087 |
| Cancer | 284 | 264 | 262 | 285 | 1089 |
| Exclusion |  |  |  |  |  |
| Diabetes | 280 | 255 | 259 | 277 | 1065 |
| Kidney disease | 278 | 253 | 254 | 279 | 1059 |
| Cancer | 280 | 253 | 255 | 280 | 1063 |
| QC warning | 289 | 262 | 265 | 290 | 1100 |
| * Three proteins (IL6, CXCL8 and TNF) were included in all four panels, resulting in a total of 1463 unique proteins. |  |  |  |  |  |

### eTable 7. Difference of 1463 proteins associated with 1-SD higher BMI in genetic analyses

Saved into an Excel file as supplementary material 

### eTable 8. List of significantly enriched biological process terms of BMI-related proteins 

Saved into an Excel file as supplementary material 

### eTable 9. List of significantly enriched biological process terms of 1463 OLINK proteins 

Saved into an Excel file as supplementary material 

### eTable 10. List of significantly enriched KEGG pathways of BMI-related proteins 

Saved into an Excel file as supplementary material 

### eTable 11. PheWAS associations of *cis*-pQTL in UKB of eight proteins showing causal relationship with BMI

| **Protein** | **rsID** | **PheWAS associations** ^**a**^ |
| --- | --- | --- |
| ITIH3 | rs9881468 | **BMI, height, impedance of leg/arm/whole body** |
| LRP11 | rs2342858 | hand grip strength, BMI |
| SCAMP3 | rs1142287 | BMI |
| NUDT5 | rs10508438 | BMI |
| OGN | rs7026361 | **height, impedance of arm/body** |
| EFEMP1 | rs59985551 | **height, trunk/body fat-free mass** |
| TXNDC15 | rs3733897 | **height** |
| PRDX6 | rs33951697 | - |

a Traits or diseases in bold: P < 5*10-8 , others were P < 5*10-6

### eFigure 1. Flow diagram of study design and participant selection in CKB

*Selection for the subcohort used simple random sampling; †Individuals may be included in more than one study arm

### eFigure 2. Distributions of individual proteins (n=1463), by OLINK panel

Separate pdf files

### eFigure 3. Volcano plots of associations of 1463 proteins with 1-SD higher BMI in observational analyses, by OLINK panel

### eFigure 4. Conventional linear associations of BMI with 20 selected proteins, by OLINK panel 

To examine the shape of the associations in observation analyses, adjusted means of proteins were calculated within each of BMI quintiles using multiple linear regression and then plotted against mean BMI within each of quintiles. Within each panel, top 5 proteins were selected including 4 positive and 1 inverse proteins.The length of y-axis represents ± 2 SD from the mean of the corresponding protein.

### eFigure 5. Volcano plots of associations of 1463 proteins with 1-SD higher BMI in conventional analyses of subcohort participants, by OLINK panel

Conventional epidemiological analyses for BMI. Results are adjusted for age, age square, sex, study area, fasting time, ambient temperature and plate ID.

### eFigure 6. Global comparisons of associations of 1463 proteins with 1-SD higher BMI in observational and genetic analyses, between men and women

### eFigure 7. Genetic non-linear associations of BMI with 20 selected proteins, by OLINK panel

Within each panel, top 5 proteins were selected including 4 positive and 1 inverse proteins. Piecewise linear method was used to calculate the estimates (adjusted for age, age squared, sex, and study area [ten groups], ascertainment, and 12 national PCs). Each line segment begins where the previous segment finished (black lines) and the intercept was set to the population mean BMI (red dot). The 95% CI in the genetic analyses are represented by the shaded patterns. The length of y-axis represents approximately ± 2 SD from the mean of the corresponding protein.

### eFigure 8. Volcano plots of associations of 1463 proteins with 1-SD higher BMI in genetic analyses, by OLINK panel

### eFigure 9. Volcano plot of associations of 1463 proteins with 1-SD higher BMI in genetic analyses of subcohort participants, by OLINK panel

Genetic epidemiological analyses for BMI. Results are adjusted for age, age square, sex, study area, fasting time, ambient temperature, and the first 12 PCs.

### eFigure 10. Correlation matrix among those adiposity-associated proteins in CKB

### eFigure 11. Human tissue-specific gene expression for the eight proteins showing causal relationship with adiposity 

Human RNA-seq data from GTEx showing the transcript per million (TPM) expression values for the genes encoding the proteins
